# Human HSPC clones balance stochastic diversification with cytokine-induced differentiation^∗^^†^

**DOI:** 10.1182/blood.2026033834

**Published:** 2026-07-30

**Authors:** Elia Colin, Dror Brook, Jonathan Izraeli, Oren Milman, Aviezer Lifshitz, Tal Bacharach, Noa Chapal-Ilani, Liran Shlush, Amos Tanay

**Affiliations:** 1 Department of Molecular Cell Biology, Weizmann Institute of Science, Rehovot, Israel; 2 Department of Computer Science and Applied Mathematics, Weizmann Institute of Science, Rehovot, Israel

## Abstract

Hematopoietic stem and progenitor cells (HSPCs) balance self-renewal with on-demand differentiation of blood lineages. Modern bone marrow atlases specify static transcriptional states, but not HSPC dynamics, which are only partly understood, particularly in humans. Here, we introduce cHSPCTrack (clonal HSPC tracker), an in vitro framework for serially tracking clonal differentiation and fate acquisition from human HSPCs extracted from circulation. By following thousands of single-cell–derived clones stimulated by different cytokine combinations, cHSPCTrack reconstructs the balance between stochasticity and directed differentiation in human HSPCs. Single HSPCs stochastically yield multiple lineages under a uniform cytokine environment, with stimulatory inputs modulating differentiation probabilities by introducing biases toward preferred fates. In parallel, differentiation rates vary stochastically across and within clones. Clonal memory stabilizes the induction of a few genes per clone over otherwise archetypal differentiation trajectories. Within clones, multiple fates can interact with each other, as shown for clones mixing basophils, eosinophils, and mast cells with other fates. cHSPCTrack opens avenues for understanding hematopoietic clonal dynamics in health and disease.

## Key Points

• cHSPCTrack uses circulating human HSPCs to reconstruct clonal differentiation dynamics.
• Human HSPC clone differentiation varies stochastically in lineage output and differentiation rate, even in controlled isolated environments.

## Visual Abstract

**Figure:**
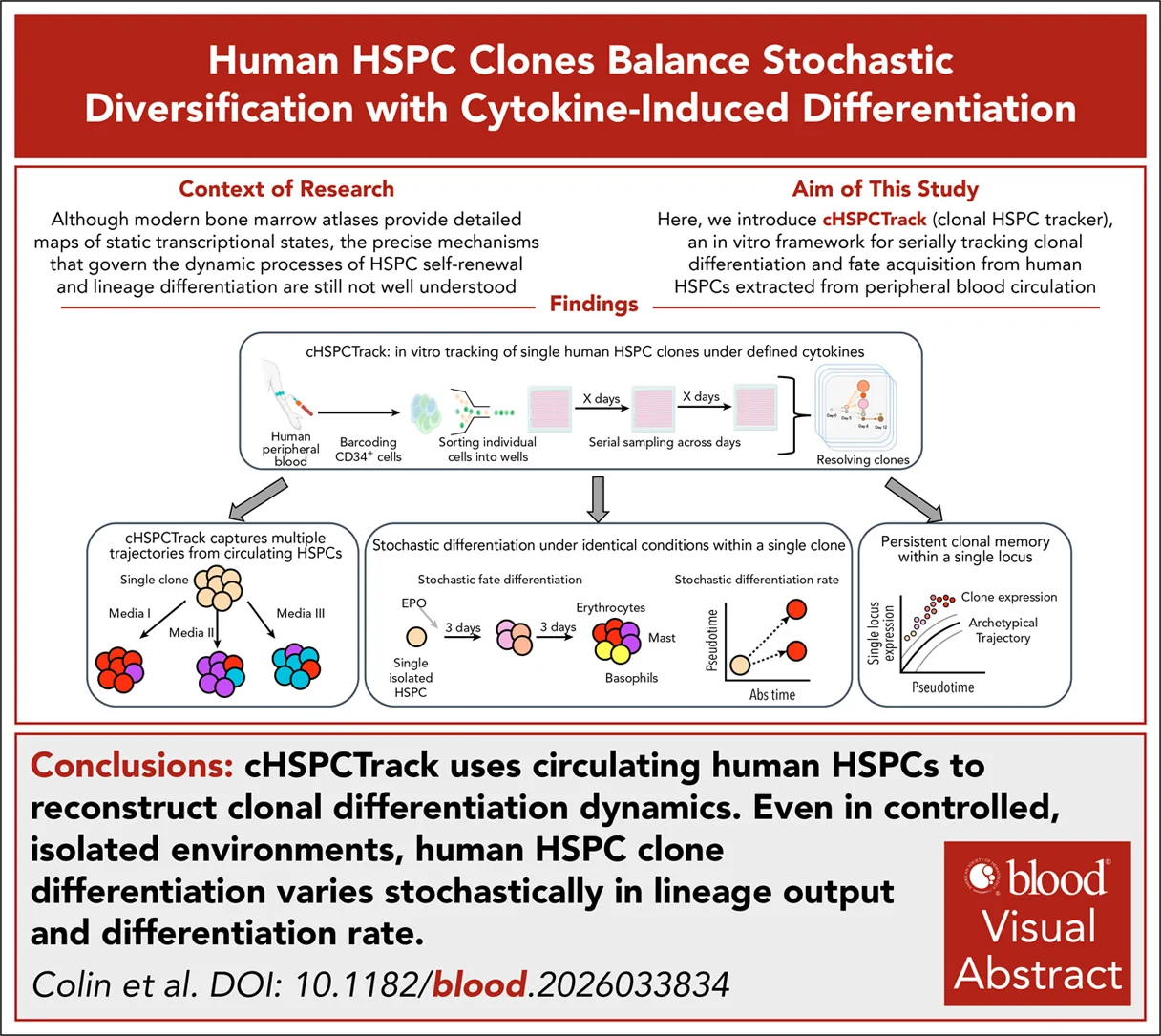

## Teaser

The mechanisms governing hematopoietic stem and progenitor cell (HSPC) self-renewal and lineage differentiation remain incompletely understood. Colin et al describe cHSPCTrack (clonal HSPC tracker), which combines ex vivo clonal lineage tracing with single-cell transcriptomics to track thousands of barcoded human HSPC clones during cytokine-driven differentiation. Their findings show that lineage commitment reflects the interplay between intrinsic stochastic clonal diversification and cytokine-mediated lineage bias.

## Introduction

Hematopoiesis is a continuous regenerative process that sustains the production of mature blood cells through the differentiation of hematopoietic stem and progenitor cells (HSPCs).^1^ HSPCs dynamically balance self-renewal and differentiation to maintain blood and immune homeostasis.^2^^,^^3^ The plasticity of HSPCs allows clones to maintain a multipotent state that, in turn, drives the production of diverse lineages on demand. Understanding how HSPC fate decisions are regulated and how multipotent cells process cytokine signals (eg, erythropoietin [EPO] for erythropoiesis or granulocyte colony-stimulating factor [G-CSF] for granulopoiesis) toward lineage restriction remains a central challenge in hematopoietic biology.

For decades, hematopoiesis has been described as a hierarchical process, in which multipotent stem cells progressively lose potential through a series of discrete, binary fate decisions. In this model, oligopotent intermediate cells act as lineage branching points that ultimately give rise to lineage-restricted progenitors and mature blood cell types.^4^, ^5^, ^6^, ^7^, ^8^, ^9^ Recent advances in single-cell transcriptomics revised this model, revealing extensive heterogeneity within phenotypically defined cell populations, and showing that differentiation proceeds along a continuum of transcriptional states rather than through discrete transitions.^9^, ^10^, ^11^, ^12^, ^13^, ^14^, ^15^ This transcriptional continuum is still assumed to be spanned by differentiating multipotent HSPC clones with dynamics driven by external signals.^16^, ^17^, ^18^ However, the specificity and determinism of the process, as well as how clones balance multipotency with dynamic fate preferences, are not well understood. In some lineages, such as basophils and eosinophils, the developmental hierarchies within the transcriptional continuum itself are not fully resolved.^14^^,^^19^, ^20^, ^21^

Lineage tracing approaches have further advanced our understanding of hematopoietic differentiation by linking transcriptional states to clonal fate outcomes, providing a powerful framework to dissect the molecular drivers of lineage commitment.^20^^,^^22^, ^23^, ^24^, ^25^, ^26^ However, most of these studies have been conducted in murine models, and the precise dynamics of lineage commitment in human HSPCs remain poorly defined. In particular, the interaction between intrinsic variability and extrinsic cues in shaping fate decisions remains unclear. Human studies often rely on static molecular snapshots that lack the dynamic and clonal resolution necessary to reconstruct fate decisions over time and cannot probe external signaling effects in a fully controlled fashion.

To address this gap, we use human circulating HSPCs isolated from CD34^+^ human blood.^14^^,^^21^ Circulating HSPCs may undersample quiescent long-term hematopoietic stem cells, but are still highly multipotent with excellent representation of myeloid lineages. This enabled us to develop cHSPCTrack (clonal HSPC tracker), a scalable in vitro platform for tracking HSPC clonal dynamics. We integrate circulating HSPC capture with high-throughput lentiviral barcoding, serial single-cell transcriptome profiling of cultured clones, and stimulation with different cytokine combinations. By tracking thousands of clones across multiple time points and microenvironmental conditions, cHSPCTrack reconciles the 2 opposite trends shaping clonal differentiation dynamics: stochasticity and bias. First, it shows that remarkable stochasticity is linked with intraclonal fate diversity, even under constant and homogeneous cytokine stimulation. Second, it uncovers the specific fates preferred by each cytokine combination, thereby reconfirming and revising the hematopoietic progenitor hierarchy. cHSPCTrack provides opportunities for the characterization of differentiation potential in normal and abnormal human hematopoiesis, to bridge static transcriptional atlases and models of hematopoietic dynamics.

## Methods

CD34^+^ HSPCs were enriched from peripheral blood mononuclear cells of healthy donors by magnetic selection and transduced with a high-complexity lentiviral CellTag library. Single green fluorescent protein–positive cells were sorted into 384-well plates and cultured under defined cytokine combinations. To capture clonal dynamics, we used a serial sampling strategy: 50% of each well was harvested at multiple time points for 10× Genomics 3ʹ single-cell RNA-sequencing (scRNA-seq), whereas the remaining 50% continued to mature. Barcode identity of cells and wells was recovered by combining targeted genomic DNA sequencing and targeted genotyping of transcriptomes, linking clones to their culture condition using a dedicated graph algorithm for imputing missing data. scRNA-seq data were organized into metacells and annotated into cell types and states as previously described.^27^ Differentiation trajectories were modeled using de novo inference of gene programs and reclustering of single cells given inferred program activities.

## Results

### cHSPCTrack: an in vitro platform for longitudinal tracking of human HSPC clones

To profile transcriptomes of differentiating human HSPC clones under defined external signals, we developed cHSPCTrack (Figure 1A). The assay starts by isolating CD34^+^ cells from the peripheral blood of human donors and transducing them with a lentiviral CellTag library comprising ∼65 000 unique barcodes^28^ (supplemental Figure 1A; available on the *Blood* website). CellTags serve as cell identifiers that are captured and reverse transcribed alongside cellular mRNA during 3ʹ scRNA-seq library preparation, enabling clonal reconstruction. Barcoded single cells are sorted into 384-well plates and treated with distinct cytokine conditions. At defined time points, 50% of the cells from each well are pooled and processed for 10× Genomics scRNA-seq, whereas the remaining 50% of the cells continue to differentiate in culture. On the final sampling day, each well is split: 50% of the cells are processed for scRNA-seq, and the other 50% are harvested for DNA extraction and amplicon-based CellTags sequencing. This strategy enables retrospective assignment of each transcriptome to its original well and culture condition (supplemental Table 1). Altogether, cHSPCTrack temporally profiles single-cell clones under controlled stimulation, setting the stage for modeling intraclonal dynamics and their modulation by cytokines.

**Figure 1.**
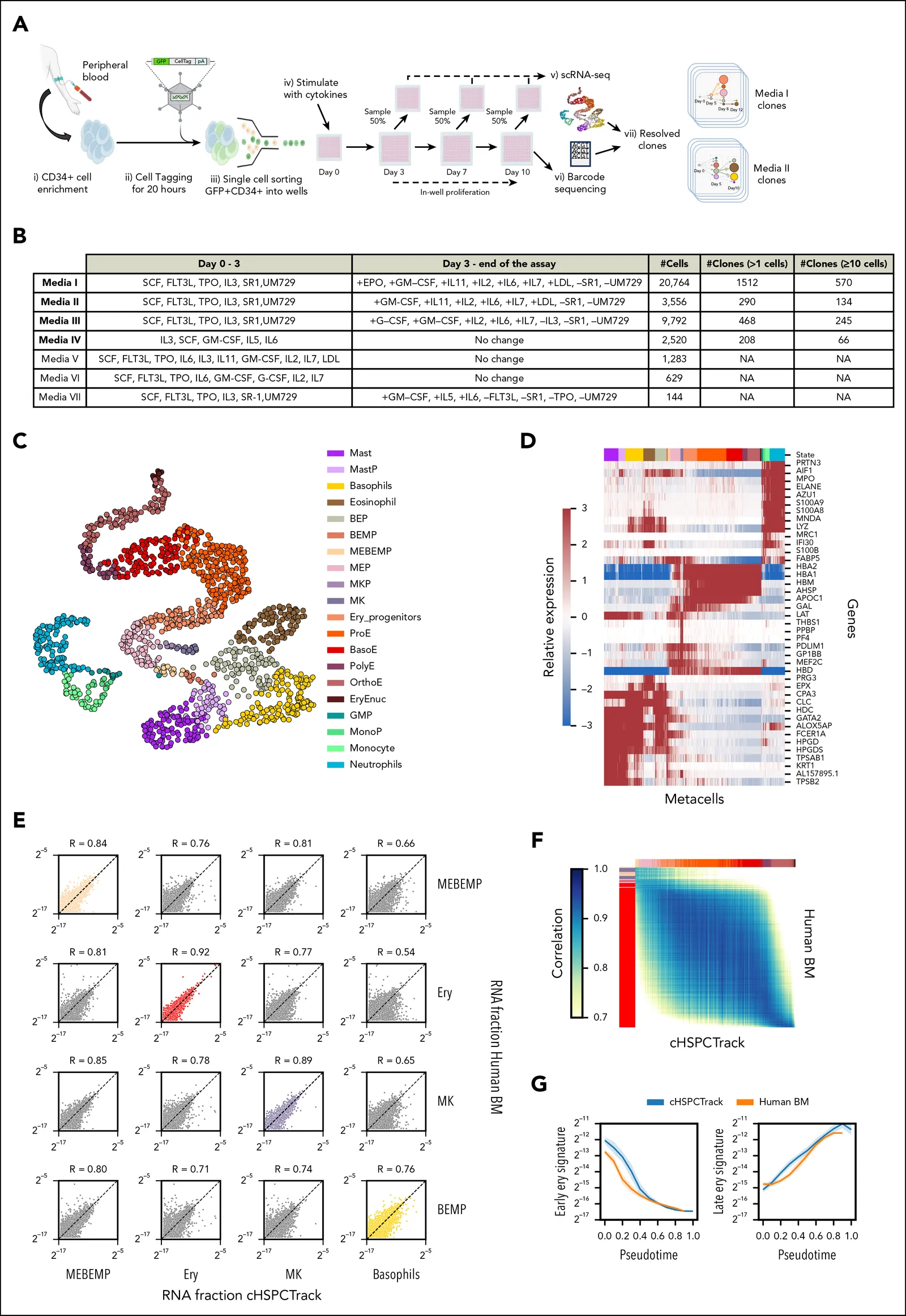
**cHSPCTrack enables longitudinal clonal tracking of human HSPC differentiation.** (A) Schematic representation of the cHSPCTrack assay and experimental design: (i) enrichment of CD34^+^ cells from human peripheral blood; (ii) lentiviral CellTag barcoding for 20 hours; (iii) single-cell sorting of barcoded cells into wells; (iv) clonal expansion under defined cytokine stimulation; (v) serial sampling of ∼50% of each well across multiple time points for scRNA-seq and barcode capturing using genotyping of transcriptomes; (vi) DNA sequencing of each well at the last time point to recover barcode composition; (vii) reconstruction of clonal structure and inference of clonal dynamics across time points. (B) Cytokine composition of the 7 media used with cHSPCTrack. The first 4 media (marked in bold) are used when presenting clonal analysis. (C) Annotated 2-dimensional uniform manifold approximation and projection of the metacell manifold. Metacell colors indicate inferred cell states and are used consistently across all subsequent panels. (D) Heat map showing the relative expression of marker genes (rows) used for cell-state annotation across cell states (columns). (E) Comparison of cHSPCTrack metacells with in vivo human BM HCA320K metacells. Each panel shows log2 of the RNA fractions for genes in 1 metacell type. (F) Pearson correlation between erythroid trajectory metacells (MEBEMP→EryEnuc) in the in vitro and BM data set. The color bar shows cell-state identities. Metacells are ordered by pseudotime in both data sets. (G) Early (left) and late (right) erythropoiesis signatures (log2 of total RNA in the respective gene modules, y-axis) across pseudotime in the in vitro and BM data set. BasoE, basophilic erythroblast; Ery, erythroid; EryEnuc, enucleated erythroid; GMP, granulocyte-monocyte progenitor; MastP, mast cell progenitor; MKP, megakaryocyte progenitor; MonoP, monocyte progenitor; OrthoE, orthochromatic erythroblast; PolyE, polychromatic erythroblast; ProE, proerythroblast.

### Tracking myeloid HSPC clonal dynamics in healthy donors

We applied cHSPCTrack to CD34^+^ cells isolated from 18 healthy donors, aged 28 to 58 years (supplemental Table 2). Cells were grown under 7 cytokine combinations (hereafter referred to as Media I-VII; Figure 1B; supplemental Table 3), focusing on the derivation of myeloid fates, as these were previously shown to dominate circulating HSPC cell populations. Labeled clones were sampled for scRNA-seq at 2 or 3 distinct time points of differentiation, collectively accumulating data on sampling days 3 to 12. After quality control and doublet removal, 67 387 single cells were retained for downstream analysis and integrated across all media conditions and all time points to construct a metacell manifold model (Figure 1C; supplemental Figure 1B). Cell subtypes were annotated based on the expression of previously reported markers (Figure 1D), showing that cHSPCTrack, with our stimulation conditions, captured the continuous differentiation landscape of myeloid lineages linking multipotent progenitors to masts, eosinophils, basophils, erythroids, megakaryocytes (MK), monocytes, and neutrophils.

### cHSPCTrack reproduces in vivo single-cell transcriptional trajectories

cHSPCTrack uses HSPCs that are a priori circulating and applies lentiviral transduction and prolonged exposure to cytokine, all of which may introduce biases compared with the native transcriptional dynamics observed in the bone marrow (BM) under physiological conditions. To test and control for such potential biases, we compared the continuous differentiation states in the cHSPCTrack-derived manifold to transcriptional profiles from several human BM data sets,^29^^,^^30^ a circulating HSPC data set,^14^ and a data set of human embryonic yolk sac and fetal liver,^31^ included due to enriched megakaryocyte representation. We confirmed that the transcriptomic states of our in vitro metacells mapped quantitatively to the corresponding in vivo states (Figure 1E). Key transcription factors (TFs) and markers were expressed in cHSPCTrack clones at similar levels compared with those observed in native BM (supplemental Figure 1C). To better understand the differentiation dynamics in our clones, we used quantitative gene signatures to infer differentiation pseudotime (supplemental Figure 1D). We initially focused on the highly represented and well-identified erythropoiesis trajectory (supplemental Table 4), comparing the ranking and gene signature expression of cHSPCTrack and BM erythroid progenitor states (Figure 1F-G). This analysis revealed robust matching of states along the entire trajectory. Comparable analyses across additional trajectories and data sets reproduced the same pattern (supplemental Figure 2A-C). Taken together, cHSPCTrack clones tolerated the protocol manipulation used for longitudinal tracking well, faithfully representing cellular states and differentiation trajectories that closely recapitulate in vivo dynamics.

### Our assay recovers differential HSPC fate biases in response to cytokine environments

The cell state composition of the acquired circulating HSPCs after labeling and before stimulation provided us with a good representation of progenitor states (supplemental Figure 3A-B). The current cHSPCTrack protocol does not retain lymphoid progenitors, as they do not get transduced with our protocol, even though these are present in circulating CD34^+^ populations (supplemental Figure 3C-D).^14^^,^^21^^,^^32^ However, the assay is strongly enriching for multipotent myeloid progenitors (megakaryocyte-erythroid-basophil-eosinophil-mast progenitors [MEBEMP], 55%) as well as more restricted progenitor states (basophil-eosinophil-mast progenitors [BEMP]^14^^,^^33^ and megakaryocyte-erythroid progenitors [MEP]). Exposure of these progenitors to different cytokine environments with cell sampling at multiple time points (Figure 2A) gave rise to distinct differentiation state compositions (Figure 2B-C), reflecting the multipotency and plasticity of circulating HSPCs. Of note, we focused our analysis on 4 media only, as the remaining 3 lack sufficient clonal coverage for analysis. Media I, which included EPO, strongly enriched for erythroid differentiation in 87% of the clones, as expected. Media II, which is similar to Media I but lacks EPO, supported a mixture of states, with 85% of clones differentiating into BEMP progenies. Media III, which included G-CSF, was the only condition that produced neutrophil-containing clones. Although these clones were rare (11%), they underwent massive expansion, making neutrophils the most abundant cell type (38% of all cells). Finally, Media IV, which includes interleukin-5, showed a balanced fate composition, with notable bias toward eosinophil fate (27% of clones). In summary, these data directly demonstrate the multipotency of circulating HSPCs and the utility of cHSPCTrack in tracking different differentiation dynamics at high resolution and in a highly controlled manner while changing only the external cytokine stimulation.

**Figure 2.**
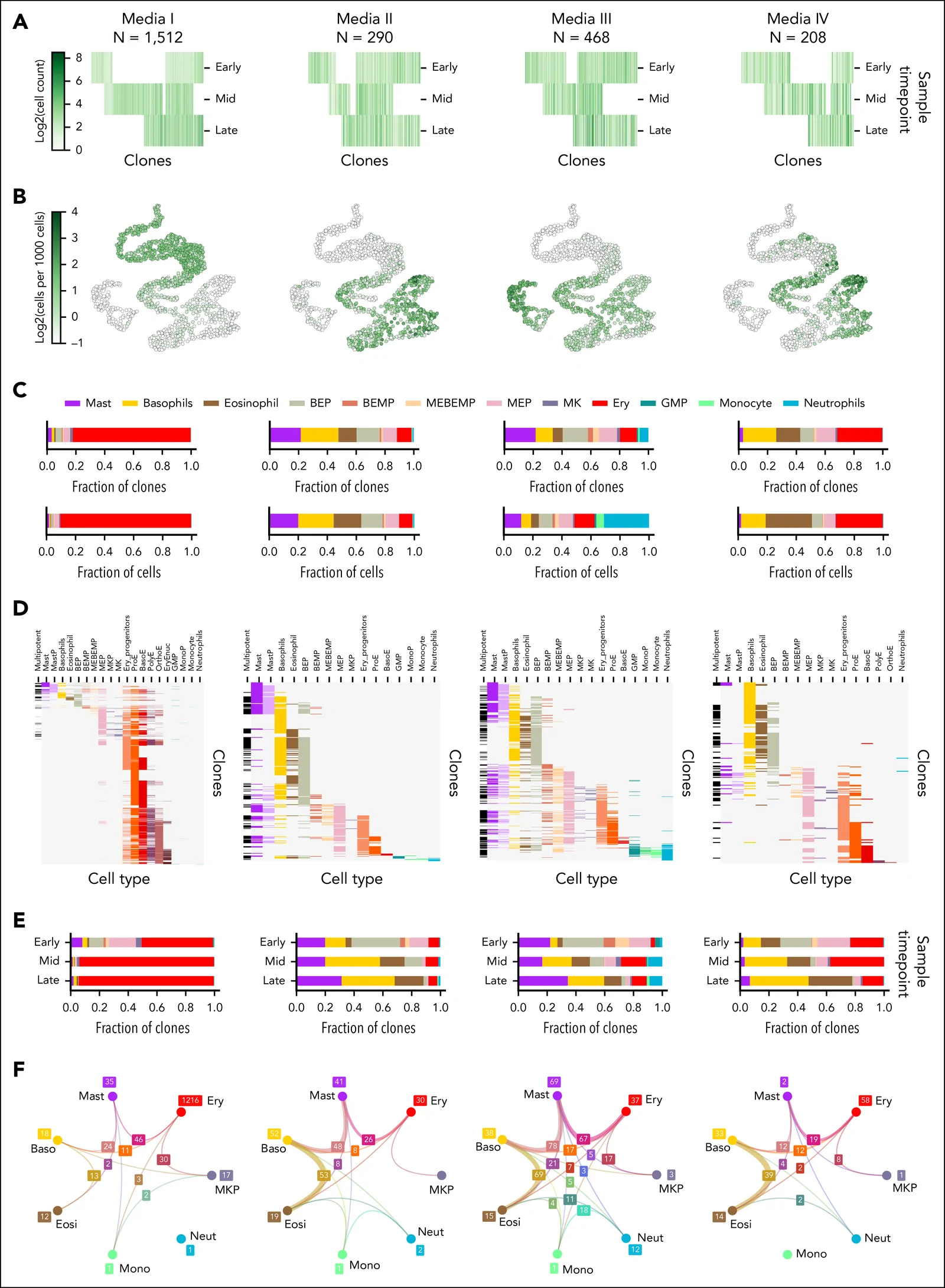
**Intraclonal stochastic differentiation under constant cytokine stimulation.** (A) Clone sampling depth is color coded across sampling time bins for each media. “N” indicates clone counts per media. (B) Distribution of cell states projected onto the metacell manifold per media (normalized per 1000 cells). (C) Cell-state fractions per media. Fraction of clones containing ≥1 cell from each state (top); overall cell-state composition (bottom). Committed progenitors are grouped by terminal fate. (D) Heat maps of cell states (columns) sampled within each clone (rows) per media (4 panels). The multipotent leftmost column indicates clones that contribute to ≥2 terminal states. (E) Fraction of clones containing each cell state per media, stratified by sampling time bin. (F) Coupling of terminal states in clones. Numbers at each node denote counts of observed single-state clones; labels on arcs denote the number of observed 2-state combinations. Edge width indicates the fraction of each combination among all combinations. The color coding of the figure is as mentioned in Figure 1. Baso, basophils; Eosi, eosinophils; Mono, monocyte; Neut, neutrophil.

### Stochastic differentiation within HSPC clones under homogeneous conditions

The observed fate biases demonstrate the remarkable diversity of circulating HSPC fates. However, in all conditions controlled cytokine stimulation still gave rise to multiple fates, which are sufficiently diverged for inference at single-cell resolution within clones. To understand whether such diversity represents clonal predispositions or intraclonal diversification, we analyzed single cells within their clonal context. Overall, cHSPCTrack assigned 44 361 cells (65.9% of total cells) to 3844 clones that contain ≥2 cells (supplemental Table 5; supplemental Figure 3E-F). Among these, 36 319 cells (53.9% of the total cells) belonged to 2188 clones that were observed across ≥2 time points of differentiation, providing longitudinal information on clonal dynamics. cHSPCTrack prioritizes serial sampling of clones and tracks short-term exposure, and we thereby relied on sampling multiple clones rather than fully covering a few large clones. We recovered >100 clones spanning ≥2 time points in each of the 4 cytokine combinations discussed above (supplemental Figure 3G). Remarkably, analysis of the distribution of cell states within single clones (Figure 2D) revealed multipotency not only between but also within clones. For example, clone O12_I_7 (Media I, n = 380) mixed erythroid and mast fates, whereas clone B9_III_6 (Media III, n = 25) combined basophil and eosinophil fates (supplemental Figure 3H). Overall, the frequency of multilineage clones depended on the cytokine environment, ranging from 7% in Media I to 48% in Media III. This suggests that specific signals can bias differentiation strongly, but not completely inhibit some capacity of single cells to adopt different transcriptional identities, even when exposed to the same cytokines. As our assay stimulates cells in isolation from their native morphological niche structures, asymmetric sister-cell behavior cannot be driven through selective interactions with the stroma or pre-existing signaling gradients. It must instead arise stochastically from intracellular regulatory variance and/or stochastic response to stimuli.

### Cytokine exposure gradually restricts intraclonal fate mapping

Although multipotency in circulating HSPC clones was observed pervasively in our data, it changed over time toward specific fate combinations in a media-specific fashion (Figure 2E; supplemental Figure 3I). In Media I, EPO exposure gave rise to massive erythroid differentiation even at early time points (days 3-5), and this was further accentuated in prolonged exposure (42% compared with 97%, *P* < .05). Interestingly, when coupling fates based on co-occurrence of cells within individual clones at late time points (Figure 2F, left), we observed mixed erythroid/mast clones (n = 46 [3.2%]), erythroid/basophil clones (n = 11 [0.8%]), and erythroid/megakaryocyte clones (n = 30 [2.1%]), indicating these alternative fates can develop at a lower rate even under EPO exposure.

Media II, which is identical to Media I but lacks EPO, failed to induce advanced (enucleated) erythropoiesis, with only 30 (11%) clones generating only erythroid output. Instead, it generated basophil/mast and basophil/eosinophil clones predominantly. Media III, which contains G-CSF, induced granulocyte monocyte progenitor (GMP)-derived proliferation and accordingly induced neutrophil/monocyte (n = 18 [4.5%]) combinations. Low-frequency eosinophil/neutrophil and eosinophil/monocyte combinations were still detected, but the majority of Media III non-GMP clones were dominated by mast/basophil (n = 78 [19%]), basophil/eosinophil (n = 69 [17%]), eosinophil/erythroid (n = 7 [1.7%]), mast/erythroid (n = 67 [16.2%]), and erythroid/megakaryocytes progenitor (MKP) (n = 17 [4%]) clones. Finally, Media IV clones exhibited a dominant emergence of basophil/eosinophil combination (n = 39 [21%]). Taken together, these results showed that each cytokine combination enriched not only for specific lineages, but also for characteristic fate combinations.

### A progenitor transcriptional program involves mixed expression of lineage-specific TFs

We and others have previously observed that, in the hematopoietic multipotent state, transcriptional regulators of multiple lineage-specific programs are already weakly expressed.^14^^,^^32^, ^33^, ^34^, ^35^, ^36^, ^37^ For these genes, differentiation involves repression in most lineages and induction only for the lineage(s) requiring the gene. We hypothesized that a similar promiscuous multipotent state defines other bifurcating progenitors, such that stochastic fluctuation of lineage regulators within these cells may help explain the intraclonal diversity reported above. To test this, we evaluated the bifurcation path of MEBEMP cells (which already likely lost the potential to generate common lymphoid progenitor [CLPs]) toward MEP or BEMP states, and the bifurcation of MEP (which likely already lost their BEMP potential) toward erythrocytes and megakaryocytes. For further control, we also evaluated the bifurcation between GMPs, monocytes, and neutrophils, although these trajectories were more difficult to resolve at high resolution in our data. As shown in Figure 3A, numerous TFs distinguish the bifurcated MEBEMP states, including all the major lineage regulators (eg, LMO4 for BEMP and KLF1 for MEP). Strikingly, the majority of these TFs show significant nonzero expression in the MEBEMP state. This can be observed in more detail in Figure 3B, where TF pairs such as GATA2/GATA1, LMO4/LMO2, and MAX/MYC transition from being coexpressed in progenitors to asymmetric fate-specific resolution. Some of these factors are further regulated dynamically in MEP and GMP progenies, for instance, MYC becomes repressed across all mature fates, whereas MAX continues to increase with MK-specific induction. Further analysis of TF expression in the MEP state suggests a similar trend but with different factors (Figure 3C-D). For example, the MK factors PBX1, RUNX1, and MEF2C, and the erythroid factors KLF1, BCL11A, and NFIA are expressed at intermediate levels in MEPs, and resolve asymmetrically as cells commit to erythroid or MK pathways.

**Figure 3.**
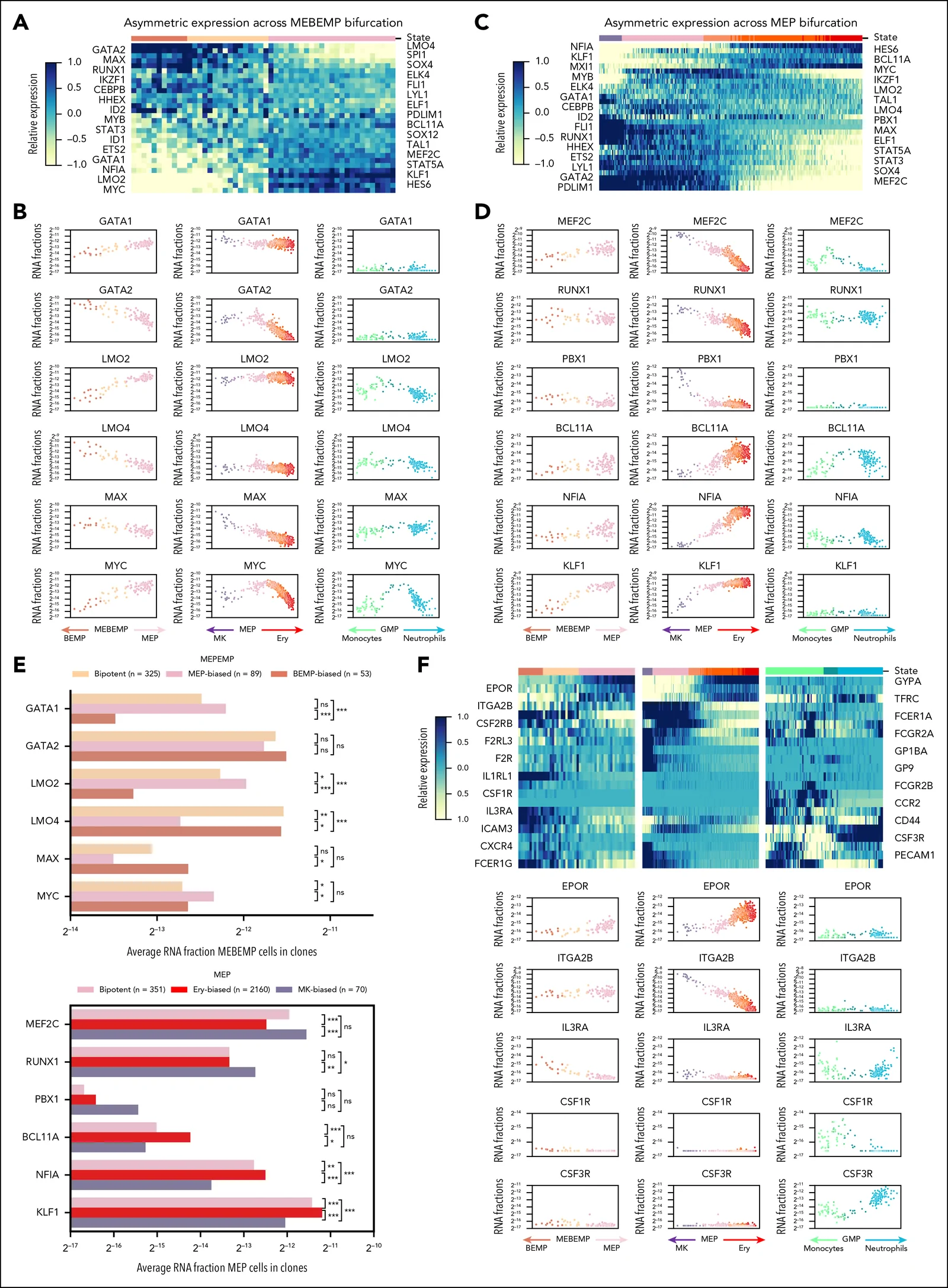
**Progenitor states coexpress TFs of competing lineages.** (A) Heat map showing expression levels of multiple TFs across BEMP, MEBEMP, and MEP metacells. (B) Selected MEBEMP branch-specific TF expression across metacells (points) in the MEBEMP (left), MEP (middle), and GMP (right) bifurcations. Each panel shows 1 TF (log_2_ fractional expression), with metacells ordered by pseudotime. (C) Heat map showing expression levels of multiple TFs across MK, MEP, and erythroid metacells. (D) Selected MEP branch-specific TF expression across the MEBEMP (left), MEP (middle), and GMP (right) bifurcations. (E) Mean normalized expression (gene unique molecular identifiers [UMIs]/total UMIs per cell) for specific TFs in progenitor states conditioned on clone output. (Top) MEBEMP TFs (B), comparing bipotent (MEP + BEMP output) vs biased clones. (Bottom) MEP TFs (D), comparing bipotent (MK + erythroid) vs biased clones. ∗*P* < .05; ∗∗*P* < .01; ∗∗∗*P* < .001. (F) Heat map showing expression levels of multiple receptor genes across progenitor states (top). Expression dynamics of select receptor genes along the MEBEMP, MEP, and GMP bifurcations (bottom).

Given these data, we next tested whether fluctuating expression levels of lineage regulators in progenitor cells are predictive of their future fates. We divided MEBEMP progenitors into MEP-biased, BEMP-biased, and bipotent classes based on the cell state of their later stage sampled sister cells. This classification is an approximation, as we are not sampling all cells in the clone and are eliminating the progenitor itself; however, we still detected significant differential expression of key regulators between the groups. For example, GATA1 and LMO2 expressions were higher in progenitors of MEP-biased clones, whereas LMO4 and MAX expressions were higher in progenitors of BEMP-biased clones (Figure 3E). An analogous effect was detected in the MEP bifurcation toward erythroid and MK states. These findings indicate that variability in the expression of lineage regulators within progenitors’ states contributes to the diversification of clonal outcomes. Similar behavior was also observed in cytokine receptor expression (Figure 3F).

### Clonal transcriptional memory screen identifies pervasive interclonal variation

Clones that are sufficiently sampled enable powerful screening for short-term transcriptional clonal memory. This can be defined as elevated or reduced expression of a specific gene for all cells within a clone, persisting throughout ≥1 differentiation trajectories. Such memory may emerge when stochastic regulation of a gene, or set of genes, is transmitted in a semistable fashion through proliferation and differentiation.^38^, ^39^, ^40^, ^41^, ^42^ To detect these events, we used our derived quantitative trajectory models as a baseline and projected the RNA profiles of each cell onto it. For each clone, we then compared its observed total expression with the expression expected from the model (refer to “Methods”). Interestingly, we observed significant events (false discovery rate <0.05) of clonal memory in all analyzed trajectories (Figure 4A-B). For example, the screen recovered a large number of BEMP clones showing clonal reduction (n = 57) or induction (n = 11) of proteoglycan 2 as well as clones with altered clonal expression of hemoglobin variants (HBG1/2; n = 42 increased; n = 339 decreased; supplemental Figure 4A). Clones with increased fetal globin (HBG1/2) showed reduced adult globin expression (HBB/HBD; supplemental Figure 4B), consistent with competition at the β-globin locus control region.^43^, ^44^, ^45^ These events were part of a general trend by which genes that are highly induced in specific lineages were more likely to show detectable clonal effects (supplemental Figure 4C). However, this should be considered in light of the reduced screen sensitivity for lowly expressed genes. On the other hand, screening for clonal skewing at the level of gene programs yielded negative results (supplemental Figure 4D). These findings suggest that transcriptional variability can be transmitted semistably across differentiation. Clonal memory is typically restricted to a single gene or locus and does not involve coordinated changes in broader transacting regulatory programs, thereby preserving the core differentiation trajectory while allowing persistent intraclonal heterogeneity.

**Figure 4.**
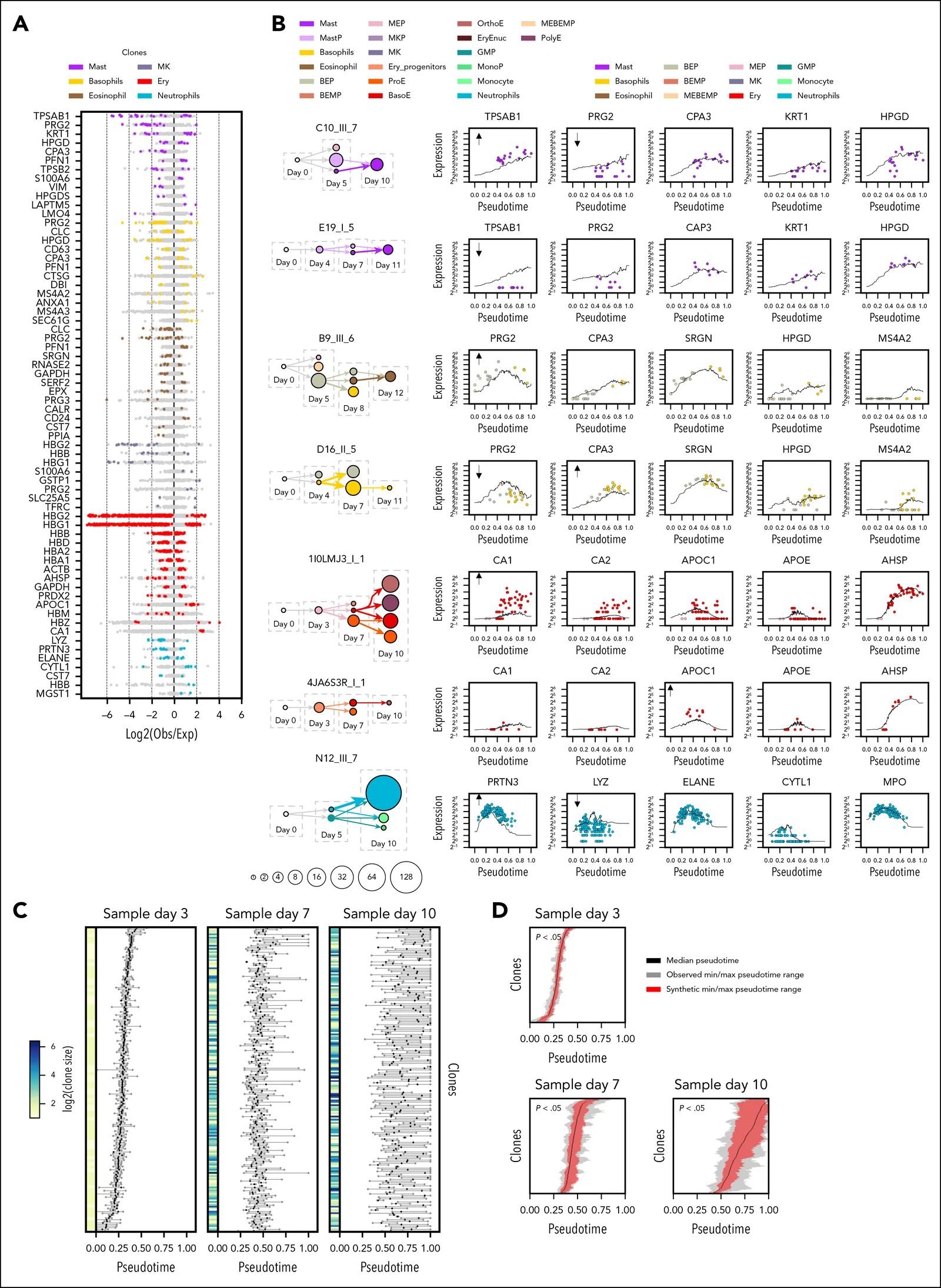
**Clones exhibit single locus transcriptional memory and stochastic differentiation rates.** (A) Top 15 clonally overexpressed or underexpressed genes across trajectories. Dots represent clones, which are colored by trajectory if significantly inducing or repressing the gene. x-axis represents log_2_(observed/expected) expression per clone. (B) Examples of clone-specific memory. Clone schematics (circle size = number of cells per state per day; clone names follow *ID_media_replicate* format) with fine-grained states (including intermediates states, legend above; left). Gene expression in down-sampled cells of each clone (log_2_); arrows mark significant overexpression or underexpression, coarse states only (progenitor vs committed, legend above; right). Trend line in black represents the mean behavior over all cells. (C) Erythropoiesis pseudotime of individual cells within clones (rows) across days 3, 7, and 10. Horizontal line in gray marks the range in each clone with a dot in black representing the median. Color-coded left column represents cell count per clone, clones ordered by day 3 pseudotime median. (D) Observed vs synthetic (refer to “Methods”) pseudotime distributions, showing significantly larger pseudotime gaps within observed clones at each sampling day.

### Intraclonal dispersion of differentiation rates

We next asked whether stochasticity also influences the rate of differentiation within a committed lineage. Focusing on the erythroid trajectory in Media I (containing EPO), we compared the pseudotime values of all cells within each individual clone. Sister cells frequently occupied different pseudotime positions, corresponding to distinct erythropoiesis states (Figure 4C; supplemental Figure 4E). Given that all cells in a clone originated from a single sorted progenitor and were exposed to identical signals, the divergence indicated that the differentiation rate is inherently stochastic. We used resampled clones to control for low-coverage biases, showing that sister cell pseudotimes are indeed significantly overdispersed (233 of 271 clones with q < 0.05; Figure 4D; supplemental Figure 4F). These results were reproduced in other trajectories with sufficiently sampled clones (supplemental Figure 4G). The mechanism underlying intraclonal variations in differentiation rates, and whether such variation is related to retained multipotency or bipotency of cells within a clone, remains difficult to resolve in our data. The data suggest that fluctuations in differentiation rates exist at the clonal level, potentially playing a role in physiological erythropoiesis. This raises new questions about factors that influence not only fate selection but also the pace of differentiation.

### cHSPCTrack delineates a BEMP differentiation pathway uncoupled from GMP states

cHSPCTrack captures a detailed basophil-eosinophil-mast differentiation process. These trajectories are largely missing from human BM atlases, which sample progenitors, whereas terminal maturation occurs in peripheral tissues. Within these trajectories, we identified 3 clear differentiated states but also a hierarchy of progenitors (Figure 5A). As expected, we detected a common progenitor state (BEMP) expressing lower stem-like and MEP-like signatures compared with the MEBEMP state, and initial build-up of lineage-specific regulators such as HPGD, LMO4, and CPA3 (Figure 5B; supplemental Figure 5A). Interestingly, we also identified basophil-eosinophil progenitors (BEP), characterized by intermediate expression of genes for basophils and eosinophils and reduced BEMP signatures (Figure 5C). BEP cells are enriched for endoplasmic reticulum and protein secretion pathway–related genes, which are symmetrically downregulated in basophils and eosinophils and are distinguished from a set of mast cell progenitors (Figure 5D). Importantly, we observed BEP at early time points, further supporting their progenitor capacity (supplemental Figure 5B).

**Figure 5.**
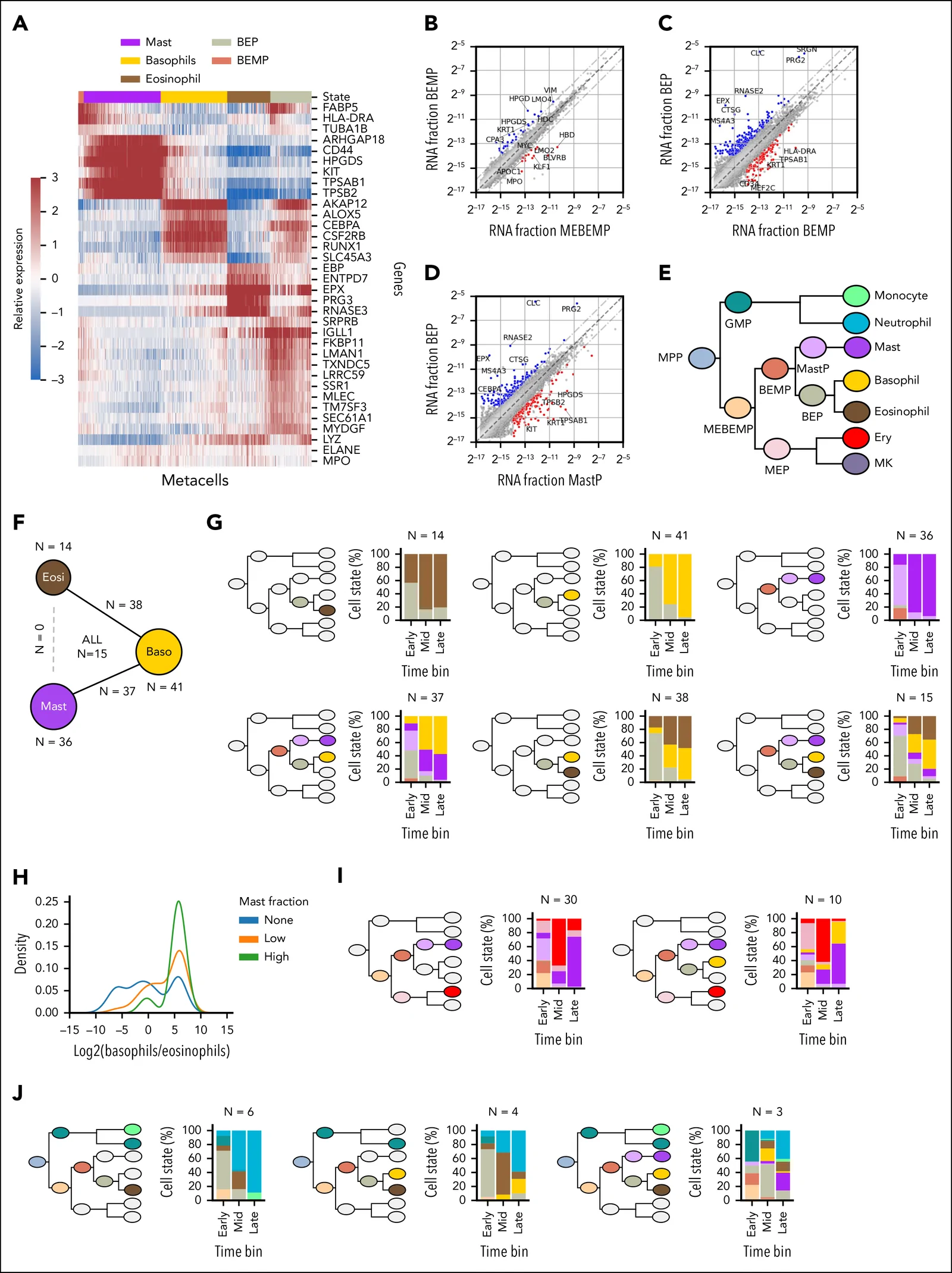
**BEMP differentiation hierarchy is defined by cytokine combinations and intraclonal interactions.** (A) Heat map of genes (rows) per BEMP trajectory cell-state metacells (columns). (B) Gene expression comparison between MEBEMP and BEMP. Each point shows the absolute expression of a gene (log_2_ UMI fraction). Diagonal dashed lines mark twofold differences. Blue/red denotes significant y-axis/x-axis overexpression. (C) Gene expression comparison between BEMP and BEP states. (D) Gene expression comparison between MastP and BEP states. (E) A schematic representation of the proposed differentiation tree for the myeloid branch of human hematopoiesis. (F) Schematic of BEMP-only clones in Media III. “N” indicates clone counts adjacent to nodes (single-fate), along edges (2-fate), and at center (all BEMP fates). (G) BEMP-only clone (Media III) differentiation paths (left) with corresponding transcriptional-state distributions over time bins (right) for each terminal state combination; single-fate clones (top), multi-fate clones (bottom). The description of “N” matches that provided in panel F. (H) Density of log_2_ basophil/eosinophil ratios, conditioned on mast-cell fraction: none (0), low (≤0.5), high (>0.5). (I) BEMP-MEP clone differentiation paths (left) with corresponding transcriptional-state distributions over time bins (right) for each terminal state combination. The description of “N” matches that provided in supplemental Figure 6E. (J) BEMP-GMP clone differentiation paths (left) with corresponding transcriptional-state distributions over time bins (right) for each terminal state combination. The description of “N” matches that provided in supplemental Figure 6G. MPP, multipotent progenitors.

To obtain a higher-resolution mapping of BEMP and BEP fates, we studied their distribution within single clones. Overall, cHSPCTrack assigned 12 849 cells to 982 clones containing ≥4 cells associated with basophil, eosinophil, or mast state. Among these, 722 (78%) contained only BEMP derivatives, 162 (16.5%) contained MEP derivatives as well, and only 37 (4%) included GMP states (n = 37). These data show that within the cHSPC state and given our specific stimulations, BEMP cells and their progenies emerge from multipotent progenitors through the MEBEMP state and not GMP-linked progenitors, consistent with previous studies (Figure 5E).^14^^,^^19^^,^^46^^,^^47^ We did note convergent induction of key GMP genes such as PU.1/SPI1, CEBPA, CEBPE, or LYZ in eosinophils and basophils (supplemental Figure 5C-D), highlighting the challenge in recovering lineage relationships through transcriptional similarity alone.

### BEMP clones display plastic combinatorial lineage output

To further understand lineage relationships in BEMP progenies, we specifically focused on clones containing BEMP-derived fates only. In Media III, we analyzed 181 clones with ≥4 such cells; 50% were composed of a single terminal state (Figure 5F), whereas 41% yielded paired outputs, either basophil-mast or basophil-eosinophil, in an equal ratio. Fully multipotent combinations accounted for the remaining 9% of the clones. Remarkably, no clones combined mast and eosinophils without including other fates. We profiled the progenitor cell population over time and inferred based on maximum parsimony the likely developmental hierarchy in groups of clones with common BEMP-derived outputs (Figure 5G). BEP frequency was high in all clone combinations containing basophils and/or eosinophils, whereas mast-only clones were enriched for BEMP cells at the early time point. This pattern supports BEP as a shared progenitor for basophils and eosinophils, contrasting with mouse models that propose a basophil-mast progenitor and separate eosinophil trajectory.^48^, ^49^, ^50^, ^51^, ^52^ These differences may reflect species- or system-specific effects and emphasize the importance of clonal tracking of primary human cells.

Media change or the presence of sister cells from the MEP or GMP lineage altered the balance between BEMP fates but did not alter their hierarchy (supplemental Figure 5E-H). In Media IV, mast-only clones were lost, and basophil-mast or multipotent clones became rare (n = 1 and n = 2, respectively), indicating that BEMP cells are skewed away from mast and toward BEP states (supplemental Figure 6A-B). Integration of all data showed that as the mast fraction within clones becomes higher, the basophil-eosinophil balance skews toward basophils (*P* < .05; Figure 5H), which suggests the possibility of intraclonal signals. Differential expression analysis further supports this as basophils and BEP cells within high-mast–fraction clones show increased expression of mast-associated genes, whereas those within high-eosinophil–fraction clones show increased expression of eosinophil-associated genes (supplemental Figure 6C-D). Furthermore, in 48 clones that mixed BEMP and MEP progenies, 30 (62%) were mast-erythroid and 10 (20%) were basophil-mast-erythroid, with other combinations being rare (Figure 5I; supplemental Figure 6E-F). This suggests erythroid cells bias BEMP toward a mast fate. In contrast, clones mixing BEMP and GMP progenies (n = 19) were enriched for eosinophil cells, implying that GMP-lineage cells bias BEMP progenitors toward eosinophil differentiation (Figure 5J; supplemental Figure 6G). Together, these results demonstrate that differentiation from the BEMP progenitor state is plastic, and can be influenced by intercellular signals even across the hematopoietic lineages, and not only through stromal or systemic cytokines.

## Discussion

In this study, we introduce cHSPCTrack, a framework for generating a high-resolution map of human hematopoietic differentiation that encompasses both clonal and transcriptional aspects. By integrating single-cell transcriptomics with lentiviral barcoding and serial clonal sampling, we tracked thousands of clones across multiple time points and culture conditions. cHSPCTrack samples human cells from circulation, capturing dynamics in myeloid progenitors; however, it is still limited in explorations of lymphoid dynamics. Serial sampling of stimulated clones for ≤12 days trades deep sampling of few clones with sparser sampling of multiple clones and tight control over the microenvironment of each clone. This enables the direct linkage of stimulation with transcriptional states and clonal outcomes, at a resolution that was so far incomplete in human HSPC studies.

Although HSPCs are known to be multipotent, our system revealed the pervasiveness of stochastic multipotency in single clones, even under identical external signals. This intrinsic multipotency is balanced by fate coupling, which makes specific terminal-state combinations recur within a clone. We show that multipotent and bipotent progenitor states are characterized by coexpression of lineage-specific TFs, thereby extending the concept of plasticity beyond the hematopoietic stem cell state. In progenitors, mixed TF expression forms a structured regulatory landscape from which alternative fates can be sampled. Within this stochastic landscape, we found that individual clones transmit semi-stable transcriptional biases across differentiation. Detected clonal memory involves a single locus and does not alter the overall lineage program. Memory events likely rely on epigenetic mechanisms such as methylation changes or mitotic feedback loops that propagate elements of the previous regulatory state to daughter cells.^38^^,^^39^ Further studies integrating epigenetic profiling, including ATAC-seq and DNA methylation, will be required to determine how those specific epigenetic features support this semistable inheritance.

Our observations highlight that progenitor output is shaped not only by intrinsic stochastic regulatory programs or global cytokine cues, but also by local, cellular interactions. For example, cHSPCTrack provides detailed reconstruction of BEMP clones and indicates that the presence of MEP- or GMP-derived sister cells can bias differentiation toward specific BEMP fates. Given this, one should remain cautious about the universality of inferences on differentiation rates and fate balances, including those provided by the present study. Although our current data support unimodal differentiation trajectories for cell states in cHSPCTrack, there may be additional intermediate states or alternative differentiation trajectories in other conditions. Importantly, cHSPCTrack interrogates lineage potential under defined ex vivo conditions. Therefore, the differentiation trajectories and lineage relationships observed here represent potentials in response to a specific cytokine environment, rather than an exhaustive map of all possible HSPC fates in vivo. Specific contexts in health and in disease may alter clonal dynamics substantially, exploiting the multipotential or bipotential of stochastically fluctuating progenitors in distinct ways. Our assay and results are thus best interpreted as providing evidence for certain mechanistically feasible clonal dynamics, rather than as an exhaustive map of all potential dynamics HSPCs may traverse. Additional sampling of circulating HSPCs from genetically varied individuals across ages and disease states will allow more comprehensive mapping of a universal fate and differentiation hierarchy.

We note that although blood is a unique and readily available source for human HSPCs in our assay, it does not completely represent the BM or other hematopoietic niches and is currently selective against lymphoid progenitors.^14^^,^^29^ On the other hand, the assay establishes a framework for interpreting how differentiation dynamics are perturbed in aging and disease. In particular, it offers a baseline against which the effects of clonal hematopoiesis and leukemogenic-associated mutations on differentiation dynamics and clonal output/coupling can be directly measured.

To conclude, we use high-resolution clonal tracking coupled with transcriptional profiling to connect static transcriptional manifolds with differentiation dynamics. This approach resolves subtle progenitor states and emergent lineage relationships that remain hard to detect in population-level analyses, refining the architecture and regulation of human myelopoiesis. Altogether, our work supports a view in which hematopoietic commitment emerges from a continuum of probabilistic trajectories: intrinsic stochastic fluctuations generate a baseline distribution of potential transitions between progenitor states, whereas cytokine microenvironments reshape these distributions. In this framework, lineage outcomes do not reflect immediate and dichotomic branching, but a modulation of probabilistic fate potential by regulatory state, environmental context, and stochastic events.

Conflict-of-interest disclosure: The authors declare no competing financial interests.

## Supplementary Material

Supplemental Methods, References, and Figures
