# Supplementary material for: Human HSPC clones balance stochastic diversification with cytokine-induced differentiation
Source: Blood. 2026 Jul 30;148(15):1880–92. doi: 10.1182/blood.2026033834 (PMC13675887; doi:10.1182/blood.2026033834)
Supplement: Supplementary file 1 [file BLOOD_BLD-2026-033834-mmc1.pdf]

## Supplemental Methods

### Human samples

Blood samples were collected from healthy adult volunteers (n = 18) recruited from the Weizmann Institute of Science (WIS) community. The cohort included both men and women (6 women and 12 men; age range: 28–58 years; See Table S2). All participants provided written informed consent permitting access to their demographic information and sequencing data (genotyping panel and single-cell RNA sequencing). The study was conducted in accordance with the Declaration of Helsinki, and all protocols were approved by the WIS Institutional Review Board (IRB protocol no. 283-1), in compliance with all relevant ethical guidelines.

### CD34+ isolation from Peripheral Blood

For each donor, 60mL of Peripheral blood (PB) were drawn into heparin tubes. Peripheral blood mononuclear cells (PBMCs) were isolated by density gradient centrifugation using Lymphoprep filled SetpMate tubes (StemCell Technology) according to the manufacturer's instructions. $CD34^+$ hematopoietic stem and progenitor cells (HSPCs) were enriched from PBMCs using magnetic CD34 beads (EasySep™ CD34 Positive Selection Kit II, StemCell Technologie), following the manufacturer's protocol. The resulting enriched fraction was counted, and typical yields were approximately 60,000 – 100,000 $CD34^+$ cells per donor.

### CD34+ transduction with CellTagging library

$CD34^+$ cells were isolated as described above and seeded into flat-bottom 96-well plates in StemSpan SFEM II medium (Stemcell Technologies) supplemented with 100 ng/mL SCF, 100 ng/mL FLT3L, 50 ng/mL TPO, 10 ng/mL IL-3, 0.75 μM SR1, and 1 μM UM729. After overnight pre-stimulation, cells were transferred to retronectin-coated wells (33 μg/mL) and incubated for 2 hours at a concentration of $1.5 \times 10^6$ cells/mL. Cells were then transduced with the CellTag-V1 lentiviral library (Addgene; containing a minimum of 19,973 unique barcodes) at a multiplicity of infection (MOI) of 30, in the presence of 1× LentiBoost (Sirion Biotech) and 8 μg/mL protamine sulfate (Sigma-Aldrich). Cells were maintained in culture overnight following transduction.

### Single cell differentiation assay

Following lentiviral barcoding, transduced $CD34^+/GFP^+$ cells were single-cell sorted into 384-well flat-bottom plates containing StemSpan SFEM II medium supplemented with various combinations of cytokines and metabolites (see Table S3). Plates were incubated at 37°C and 5% $CO_2$, and differentiation was monitored at 2 or 3 different time points after sorting (for instance day 3, 7 and 10)

At each time point, half of the culture volume from each well was collected using an automated Tecan liquid handling system and pooled for single-cell RNA sequencing (scRNA-seq) using the 10x Genomics platform. The removed volume was immediately replaced with an equal volume of fresh adequate medium to maintain culture conditions.The remaining cells in each well were maintained in culture to continue the differentiation protocol. On the last day of sampling, half of the remaining cells were harvested for scRNA-seq, and the remaining half were used for genomic DNA extraction in order to recover CellTag barcode sequences from each colony, enabling linkage of clonal identity to the original environmental condition and transcriptomic profiles.

**scRNAseq**

ScRNA libraries were processed using the Chromium Single Cell 3' Library & Gel Bead Kit V3.1 (10x Genomics). All cells were derived from in vitro differentiation of CD34+ derived from peripheral blood. Approximately 20.000 cells per sample were loaded into each channel for GEMs generation. After reverse transcription and cDNA amplification, sequencing libraries were prepared according to the 10x genomics guidelines. Libraries were then sequenced on NovaSeq 6000 Illumina sequencer.

**Genotyping of Transcriptomes (GoT)**

As recovery of CellTag barcodes directly from 10x Genomics 3′ scRNA-seq libraries was insufficiently efficient, we used a Genotyping of Transcriptomes (GoT) methods to improve barcode detection.

An aliquot of 4 µL from the amplified 10x Genomics 3′ cDNA library was used as input for targeted enrichment of CellTag barcodes via a nested PCR strategy.

<u>**Initial PCR (PCR 0):**</u>

The first PCR employed a CellTag-specific forward primer and a reverse primer

complementary to the partial Illumina Read 1 sequence to amplify the CellTag region. Following PCR amplification, products were purified using a 1.5× SPRI bead cleanup.

**Second PCR (PCR 1):**

The purified amplicons were subjected to a second PCR using a biotinylated forward primer containing a partial adapter handle and a reverse SI-PCR primer to further amplify the targeted locus. PCR products were again purified with a 1.5× SPRI bead cleanup.

**Biotin Pulldown:**

Biotinylated PCR products were captured using streptavidin-coated M-280 magnetic beads (Thermo Fisher Scientific) to enrich the targeted amplicons.

**Sample Indexing PCR:**

Enriched amplicons were amplified in an on-bead PCR using an indexed RPI-X primer (incorporating an 8 bp sample barcode) and a generic P5 primer to add full Illumina adapter sequences.

A final 1.5× SPRI bead cleanup was performed to purify the completed libraries.

Full primer sequences, including adapter and indexing primers, are listed in Supplementary Table X.

Final libraries were quantified and sequenced on an a NovaSeq sequencer together with the gene expression 10x libraries.

**CellTag Barcode Recovery by Targeted DNA Sequencing**

Genomic DNA was extracted from each colony by alkaline lysis: briefly, cells were pelleted (1300 rpm, 10 min), resuspended in 50 µL of 50 mM NaOH, heated to 99 °C for 10 minutes, cooled on ice, and neutralized with 5 µL of 1 M Tris-HCl pH 8.0. 1 µL of the resulting lysate was used as template for the first PCR.

All libraries were generated using a two-step PCR protocol. In the first PCR, target-specific primers with partial Illumina overhangs were used to amplify the CellTag region from genomic DNA of individual colonies using UltraII Q5 Master Mix (NEB) with a final primer concentration of 0.5 µM each. PCR conditions were as follows: 98 °C for 30 s; 5 cycles of

98 °C for 10 s and 60°C for 15s, followed by 23 cycles of 98 °C for 10s and 65°C for 30s and a final elongation at 65 °C for 5 min.

The first-round PCR product was diluted 1:1000 in nuclease-free water and used as input for the second PCR, which added Illumina sequencing adapters and sample-specific dual index. This second PCR was performed under the same cycling conditions and with the same concentration of primers.

Barcoded second-round PCR products were pooled in equal volumes. Libraries were cleaned and size-selected using a three-step procedure: (1) SPRI bead selection at 0.75× ratio to remove small fragments; (2) 2% agarose gel electrophoresis migration and gel extraction (Qiagen) of the correct amplicon band; and (3) a final 0.75× SPRI bead cleanup.

Final libraries were sequenced on an Illumina MiniSeq platform using 2 × 150 bp paired-end reads. All primer sequences are listed in Table X

**Genotyping and demultiplexing**

Genomic DNA was extracted from 100uL of PB from each donor using the Dneasy blood and tissue kit (Qiagen). For each experiment, DNA was extracted from individual samples prior to pooling. Three to four donor samples were pooled immediately afterward, and CD34+ enrichment was performed on the entire pool of PBMCs.

A custom genotyping panel was used for targeted DNA sequencing, designed specifically to capture polymorphic sites that are prevalently expressed at the RNA level across all cell types in our dataset. This enabled sample demultiplexing based on individual-specific SNP combinations and replaced prior antibody-based multiplexing approaches.

To demultiplex pooled 10x libraries, we used Vireo (v0.5.7) to assign cells to individual donors based on expressed SNPs. Donor identities were matched to genotypes obtained from the targeted DNA panel, which includes >2,000 SNVs selected from the 1000 Genomes Project (minor allele frequency ≥5%), excluding those in repetitive elements or sex chromosomes. Only SNPs with a minimum coverage of ≥10× in RNA and ≥20× in DNA were used for matching. The genotyping panel was designed with MIPgen to ensure high capture uniformity and specificity, and prepared by LC Science. Sequencing was performed on an Illumina NovaSeq instrument with a minimum depth of a million paired-end reads per DNA sample. Variant calling was performed using VarScan, as previously described.

**ScRNA-seq processing**

We processed fastq files by executing cell-ranger (v.6.1.2) with an hg-38 reference genome. We filtered out cells with at least 15% mitochondrial and MALAT1 expression, then removed mitochondrial and ribosomal genes and further filtered cells with ≤500 UMIs.

**Doublet detection**

We performed several steps to assign cells to individuals and to detect doublets. Our pipeline included the following steps:

1. Demultiplexing cells and calling doublets based on SNPs found in the scRNA-seq data
2. Building a metacell model using all cells, including cells marked as doublets, identifying and removing metacells made of doublets
3. Building the final metacell model with cells from all libraries and marking metacells as doublets made of doublets.
4. Identifying doublet metacells based on expression of marker genes

In the first step, we identified doublets and assigned cells to individuals using Vireo v.0.5.8[1] and Souporcell v.2.5[2] , which cluster cells based on SNPs found in sequenced RNA molecules. We executed Vireo (preceded by running cellsnp-lite v1.2.2[3]) and Souporcell on each library separately. Both methods used SNPs from our genotyping panel. We observed high agreement in doublet calling between the two methods.

A metacell model was constructed using Metacell2[4] with a target metacell size of 50 cells, including cells previously identified as doublets. Metacells containing at least threefold more doublet-marked cells than expected from the overall doublet rate were classified as doublet-enriched. All cells that belonged to a doublet metacell were then marked as doublets. We then built an additional metacell model from all libraries, performing the same process of doublet detection. Next, we marked all metacells that expressed key markers of distinct cell types as doublet metacells, further removing those cells. Finally, we built an additional metacell model (see below), without cells that were marked as doublets.

**Constructing an initial metacell model.**

We used metacells[4] with a target metacell size of 96 cells and a target of 320K UMIs per metacell, yielding 1,116 metacells. We removed ribosomal protein RNA from count matrices to control for their variability among cell types and batches. We marked histone, cell-cycle, sex-linked, and stress response genes as lateral genes. Genes with high variance (“bursty”) distributions, including NEAT1, GDF15, UBE2C,HIST1H1B, MT2A, HIST1H1D,IGFBP5, TOP2A,ASPM, CENPF,CENPE, CCNB1, HMMR, HIST1H4C, HLA-DRA, CYP1B1, PRG2, Sex-linked, and globin genes, were further flagged as noisy. Note that lateral genes are not used for inferring metacells, but are included in all downstream analyses. Metacells were

annotated based on canonical marker expression. This was used to define terminal states as Erythrocytes (HBA, HBB high), Megakaryocytes (PF4 high), Mast (KRT1, TPSAB1 high), Basophils (CPA3, CLC high), Eosinophils (EPX, CLC high), Neutrophils (CST7, PRTN3 high, VCAN low), and Monocytes(MRC1 or VCAN high, CST7 mid). From terminal states we inferred a coverage of the metacell manifold into differentiation *trajectories*: MEBEMP-MEP-Ery, MEBEMP-MEP-MK, MEBEMP-BEMP-Mast, MEBEMP-BEMP-BEP-Basophils, MEBEMP-BEMP-BEP-Eosinophils, GMP-Monocytes, GMP-Neutrophils.

### Identifying gene modules and signatures per trajectory

For each trajectory (Basophils, Mast, Eosinophils, Ery, Mk, Monocytes, Neutrophils), we used a set of marker genes taken from the literature to group metacells into 4-8 metacell clusters. These clusters represented, in all cases, stages of trajectory differentiation, which we ordered by the expression of late and early trajectory genes. We note that this order was always consistent with the mean sampling day of cells in each metacell group, and so we denote the metacell groups as *trajectory bins*. We screened for increased or decreased expression (log fold change ≥0.5 and log2 expression levels ≥-13) for each pair of consecutive trajectory bins, thus defining two gene modules for each pair. Note that modules per trajectory or between trajectories could reuse the same genes. We computed the signature expression for each gene module by summing the UMIs for the respective genes in each metacell. Modules with strong enough signal (mean log2 expression levels ≥ -7) in one (or both) of the pair bins were used in cells regrouping into clusters to ensure robust signal separation in single cells.

### Regrouping cells into clusters using gene module signatures

We filtered out cells with low UMIs count (<1024) and calculated the signature expression of each gene module in downsampled cells (n=1024). We grouped cells using K-means over the gene module signatures, aiming for 50 cells per cluster (K = 1,200). Clusters with more than 100 cells were split into two. Metacells were defined using those clusters, with gene expression profiles calculated with the collect_metacells function on the original non-downsampled cells. The resulting metacells were re-annotated based on known markers and gene modules. Note that the identification of gene modules and regrouping cells into clusters was repeated on the refined metacells to obtain the final reference model used for downstream analyses.

This procedure ensures that cell clustering reflects gradual transcriptional transitions along differentiation trajectories rather than discrete state boundaries, capturing continuous developmental progression within each system and providing gene modules for the differentiation of those trajectories.

### Pseudotime inference per trajectory

For each trajectory, trajectory-associated metacells were projected onto a PCA-reduced space applied to the matrix of gene module signatures. A principal-curve model was fitted to this PCA space. The resulting arc-lengths were normalized to [0,1], yielding a continuous pseudotime per trajectory. Cell-level pseudotime was calculated by projecting each cell onto the fitted curve using the downsampled gene module signatures.

### In vivo atlases used for comparison

We used four in-vivo datasets for comparison purposes: the Human Cell Atlas (HCA) BM dataset[5] , a $CD34^{+}CD38^{-}$ bead-based enriched BM dataset[6] , a fetal liver and yolk sack dataset[7] and a circulating HSPCs (cHSPC) cd34 bead-based enriched dataset[8]. For all models except cHSPC, we constructed a metacell model using metacell2 in a manner similar to that described above and annotated those models based on known markers. For the cHSPC dataset, we used the existing metacell model. Of note, early progenitor states were consistently represented across all *in-vivo* references, whereas later states were only partially captured or absent, depending on the coverage of each dataset. In particular, the absence of BEMP progenies in *in-vivo* datasets due to their maturation in tissues, prevented effective in-vivo/in-vitro comparison for these lineages.

### Quantitative comparison of in-vivo and in-vitro cell states

Comparison of individual states (Fig. 1D) was performed using the annotation of the in-vitro model and the HCA human BM model. Correlations between cell groups were computed over genes' log-fold expression levels, excluding lateral genes.

### Projection of in-vivo onto in vitro manifold

We filtered a union of highly expressed and variable genes of the two models (minimal expression difference of 5 $log_2$-fold between the top and bottom 1% of metacells in each model). Lateral and noisy genes were excluded. Correlations between models were calculated over the log-expression values of these genes, and the best match for each in-vivo metacell was used as for the projection over the UMAP.

### Comparison of trajectory dynamics

We projected each in-vivo atlas onto the in-vitro manifold as described above. In-vivo metacells were first filtered to retain only those exhibiting a correlation of at least 0.7 with at least one in-vitro metacell. High-confidence correspondences were defined using a reciprocal criterion: an in-vivo and an in-vitro metacell were considered matched if each appeared among the top 20 highest-correlated partners of the other. In-vivo metacells that reciprocally matched

an in-vitro metacell were considered part of the corresponding trajectory. We calculated gene module signatures and pseudotime for each reference atlas as described above. This pseudotime and matching were used for the relevant figures (Fig. 1F-G, Supp. 2B-C).

### Characterization of the baseline CD34+GFP+ composition

To define the baseline composition of the starting population used in the single-cell differentiation assays, $CD34^{+}$ cells were transduced as described above and the $CD34^{+}GFP^{+}$ fraction was bulk-sorted and immediately processed for 10x scRNA-seq. This dataset provided a reference for the initial heterogeneity and lineage distribution of the input compartment.

A metacell model for this dataset was constructed with the same lateral and bursty genes as described above. We projected the metacells onto the cHSPCTrack manifold using the same gene modules employed in building the cHSPCTrack manifold. Each metacell was assigned to the reference metacell with the highest correlation, and cell annotations were transferred accordingly based on this mapping.

### Tag extraction from amplicon data

Amplicon reads were processed to extract well-specific barcode tags flanked by a fixed prefix (CCGGT) and suffix (GAATTCG), with an 8-bp variable region between them. Reads from all FASTQ files were scanned, and tag lists were compiled per well.

Tags were counted per well, and low-abundance tags (<16 reads) were merged with the nearest tag within a Hamming distance of 1 to correct sequencing errors. After merging, valid tags were defined through a two-step filter controlling for PCR bias. For each well, the most and second-most abundant tags were identified; a tag was retained only if its read count exceeded both a global threshold (≥16 reads) and at least one-sixteenth of the second-most abundant tag in that well. This ensured the removal of PCR artifacts while preserving genuine multi-tag wells.

Tag-well score was defined by their uniqueness across wells (1 / number of wells containing the tag). Wells with fewer than 2 tags with enough reads were removed from downstream analysis. Tags appearing in a high number of wells (>10) were excluded from downstream clone–well matching analyses.

### Tag extraction from RNA data

Tag sequences were extracted from the aligned GoT BAM files by identifying reads containing the expected barcode structure flanked by the prefix (CCGGT) and suffix (GAATTCG), with an 8-bp variable tag region. Each tag was assigned to its corresponding cell and UMI using the 10x cell barcode.

Within each cell, multiple tags associated with the same UMI were collapsed by selecting the tag supported by the highest read fraction (at least ≥0.75 of reads for that UMI and tag). Tags supported by fewer than 2 reads per UMI were excluded. For each tag within a cell, only tags with at least 10 UMIs were considered valid. Non-valid tags were merged with valid tags in cases where the hamming distance was 1, correcting for single-base sequencing errors. Notably, this had little impact on subsequent clone calling. After merging, tags with fewer than 10% UMIs compared to the most abundant tag were removed, as they are likely explained by ambient noise. Cells with at least 2 tags were used in downstream clonal calling analysis.

**Classifying cells into clones**

We clustered cells into clones through a three-step process:

1. Computing a cell–cell tag similarity score.
2. Building a combined cell–cell–well graph.
3. Pruning the resulting network to obtain well-defined clone components.

For each pair of cells i,j, we calculated two directed similarity values: the fraction of UMIs in cell i explained by the tags of cell j (including tags within a Hamming distance of ≤1), and vice versa. Multiplying these two fractions produced a symmetric similarity score that reached high values only when both cells mutually explained each other's tag UMIs.

Using the tag–well scores defined above, we quantified the association between single cells and wells based on shared barcode tags. Each tag contributed to the cell–well matching score proportionally to its tag–well score, which reflects the inverse frequency of that tag across wells. For every cell–well pair, the matching score was computed as the sum of tag–well scores over tags that were both observed in the cell and assigned to the given well. Tags excluded during amplicon or RNA filtering were not considered. The resulting cell–well score matrix provides, for each cell, a quantitative measure of support for assignment to each well based on weighted overlap of barcode tags. For every cell, we identified the best and second best wells and evaluated the confidence of the assignment. A cell was linked to its best scoring well only if this well passed multiple thresholds: the matching score was at least twice as high than the second-best; the fraction of the cell's UMIs explained by the well was at least 0.5 or 1.5× higher than that of the next best; and the tag fraction of the cell's UMIs explained by the

well was at least 0.5 or 1.5× higher than that of the next best. When these thresholds were met, and the best score exceeded 1.5, or matched the theoretical best possible score for that cell while surpassing a minimal floor of 0.5, we defined a connection between a cell node and the well node.

The resulting graph combined these confident cell–well edges with the cell–cell edges. In experiments lacking amplicon information, only the cell–cell connections were used, and clones were defined directly from connected components of that graph.

We then pruned the network to eliminate ambiguous or conflicting links. Cell–cell edges connecting cell components assigned to different wells were removed to prevent artificial multi-well components. For cells without direct well links, we tested "clone compatibility" by measuring how well their tag–UMI profiles could be explained by the tags of well-linked clone members. Cells were retained only if this compatibility exceeded 0.7 and was at least 1.5× stronger than any alternative.

The final clones were defined as the connected components remaining after pruning, each representing a coherent group of cells sharing highly similar tag profiles consistent with a single well of origin, with all thresholds applied consistently across experiments.

**Clone fate coupling maps**

To generate terminal clonal fate maps (Figure 2F), we selected clones represented by at least two cells in a given condition. For each condition, we counted the number of cells per terminal fate within each clone. Clones contributing cells to a single fate only (≥2 cells) were counted as single-fate clones and their number is displayed as labels next to the corresponding node. Clones contributing to multiple terminal fates were treated as multipotent, with edge width and numeric labels proportional to the number of such clones. Because a single clone can contribute to several terminal fates, the same clone can be represented on multiple edges.

**Bifurcation metacell shared pseudotime**

To generate a shared pseudotime across bifurcations, we calculated pseudotime for the bifurcating progenitor metacells shared using the combined gene modules of both lineages (as described above). The resulting progenitor pseudotime was then concatenated with the precomputed pseudotime of the two downstream trajectories according to their relative ordering along the shared differentiation axis.

**Transcription factor expression in progenitor states**

MEBEMP and MEP progenitors were classified by clone output. MEBEMP were divided into three groups: bipotent MEBEMP (clones contributing to both MEP and BEMP lineages), MEP

biased (clones restricted to MEP, Ery, or MK states), and BEMP biased (clones restricted to BEMP, BEP, Mast, Basophil, or Eosinophil states). MEP were similarly divided into bipotent MEP (Ery + MK), Ery biased, and MK biased groups.

For each group, normalized expression values were obtained from single MEBEMP or MEP cells and averaged across cells per transcription factor. Statistical differences in TF expression between groups were assessed by chi-square tests on total UMI counts per gene, with multiple-testing correction using the Benjamini–Hochberg procedure.

**Identification of clonal memory genes**

For each trajectory, we kept clones with ≥8 cells and downsampled every cell to 2,048 UMIs. We built an internal expectation by sampling each cell from its matched metacell profile in a multinomial fashion and then summing expected counts within each clone. Observed counts were the downsampled cell counts summed per clone.

Per clone and gene, we first asked if the cell-level deviations from expectation are consistent: paired Wilcoxon on observed vs expected cell counts within the clone at $p<0.01$, applied only to genes with total UMIs in clone ≥8 in either observed or expected, and ≥4 UMIs in at least 20% of cells in either. For these candidates we then tested the clone-level excess using a Pearson $\chi^2$ test on clone-summed counts, with Benjamini–Hochberg correction across all tested pairs and a threshold of $q<0.05$. We required direction consistency: the sign from clone-summed counts had to match the majority sign across cells, where a majority means ≥75% of non-tied cells and a minimal effective sample of max(3 cells, 5% of the clone cells), capped at 10 cells.

Pairs that satisfied all three—Wilcoxon $p<0.01$, $\chi^2$ $q<0.05$, and direction agreement—were considered clonal memory genes. For figure 4A we remove the predefined lateral genes, removed Monocyte trajectory clones due to low clone counts, and kept only genes recurring in more than one clone per trajectory, and showed the top 15 per trajectory.

**Calculating intra-clonal dispersion of differentiation rates**

For each trajectory, analyses were limited to the condition containing the highest clone count (I for erythropoiesis; III for all others). Pseudotime was assigned by projecting cells onto the precomputed metacell principal curve after downsampling each cell to $2^{11}$ UMIs; cells below this threshold were excluded. Clones represented by fewer than two cells in at least two time points were removed, as were GMP and MK trajectories with insufficient clonal coverage. All subsequent analyses used this filtered set of cells and clones.

To quantify clone-specific variability in differentiation rate, we generated 1,000 synthetic replicate datasets by UMI-level resampling. For each clone–day pair, downsampled UMIs were aggregated into a pseudobulk expression profile, from which synthetic single-cell profiles were sampled without replacement, preserving the observed cell count per clone–day and ensuring each UMI was used once. For each synthetic cell pseudotime was calculated as described above. These pseudotime distributions were used to derive per-clone statistics of differentiation rate and temporal dispersion. Clone-level p-values were computed from the 1,000 synthetic runs and adjusted using the Benjamini–Hochberg method. The global deviation between observed and expected pseudotime spacing was assessed using a Kolmogorov–Smirnov test.

Main figures display erythroid clones sampled at three time points. Supplementary figures show two-time-point clones only, due to low three-time-point sampling in media III.

**DE analysis between states or clone coupling**

To compute differential expression between states or between subsets of cells within the same state but differing in clonal coupling, we aggregated all cells matching the specified criteria. Expression values from all cells in a group were summed to generate a pseudobulk profile. This profile was normalized to a total of 1 and transformed using $\log_2$(value). For each gene, a p-value was computed using a chi-squared test and adjusted for multiple testing with the Benjamini–Hochberg method. Scatterplots display all genes, with blue points denoting genes significantly overexpressed in the y-axis group and red points denoting those significantly overexpressed in the x-axis group.

**Analysing BEMP-related clones**

For the identification of BEMP-related clones, we first restricted the analysis to clones with reliable clone information of at least four BEMP lineage cells (BEMP, BEP, Basophils, Eosinophils, or Mast). All analyses were performed separately for each media.

We constructed a clone-by-cell-type count matrix. We derived three groups for downstream analyses: (i) BEMP-only clones, whose output was confined to the BEMP compartment; (ii) BEMP–erythroid clones, which additionally contained erythroid cells; and (iii) BEMP–GMP clones, which additionally contributed to the GMP compartment. Clones were further stratified by their mature BEMP fate combinations (basophil-only, eosinophil-only, mast-only, and all pairwise and triple combinations of basophil/eosinophil/mast).

## REFERENCES

1. Huang, Y., McCarthy, D. J. & Stegle, O. Vireo: Bayesian demultiplexing of pooled single-cell RNA-seq data without genotype reference. *Genome Biol.* **20**, 273 (2019).
2. Heaton, H. *et al.* Souporcell: robust clustering of single-cell RNA-seq data by genotype without reference genotypes. *Nat. Methods* **17**, 615–620 (2020).
3. Huang, X. & Huang, Y. Cellsnp-lite: an efficient tool for genotyping single cells. *Bioinformatics* **37**, 4569–4571 (2021).
4. Ben-Kiki, O., Bercovich, A., Lifshitz, A. & Tanay, A. Metacell-2: a divide-and-conquer metacell algorithm for scalable scRNA-seq analysis. *Genome Biol.* **23**, 100 (2022).
5. A single cell immune cell atlas of human hematopoietic system - Overview - HCA Data Explorer. https://explore.data.humancellatlas.org/projects/cc95ff89-2e68-4a08-a234-480eca21ce79.
6. Setty, M. *et al.* Characterization of cell fate probabilities in single-cell data with Palantir. *Nat. Biotechnol.* **37**, 451–460 (2019).
7. Wang, H. *et al.* Decoding Human Megakaryocyte Development. *Cell Stem Cell* **28**, 535-549.e8 (2021).
8. Furer, N. *et al.* A reference model of circulating hematopoietic stem cells across the lifespan with applications to diagnostics. *Nat. Med.* **31**, 2442–2451 (2025).

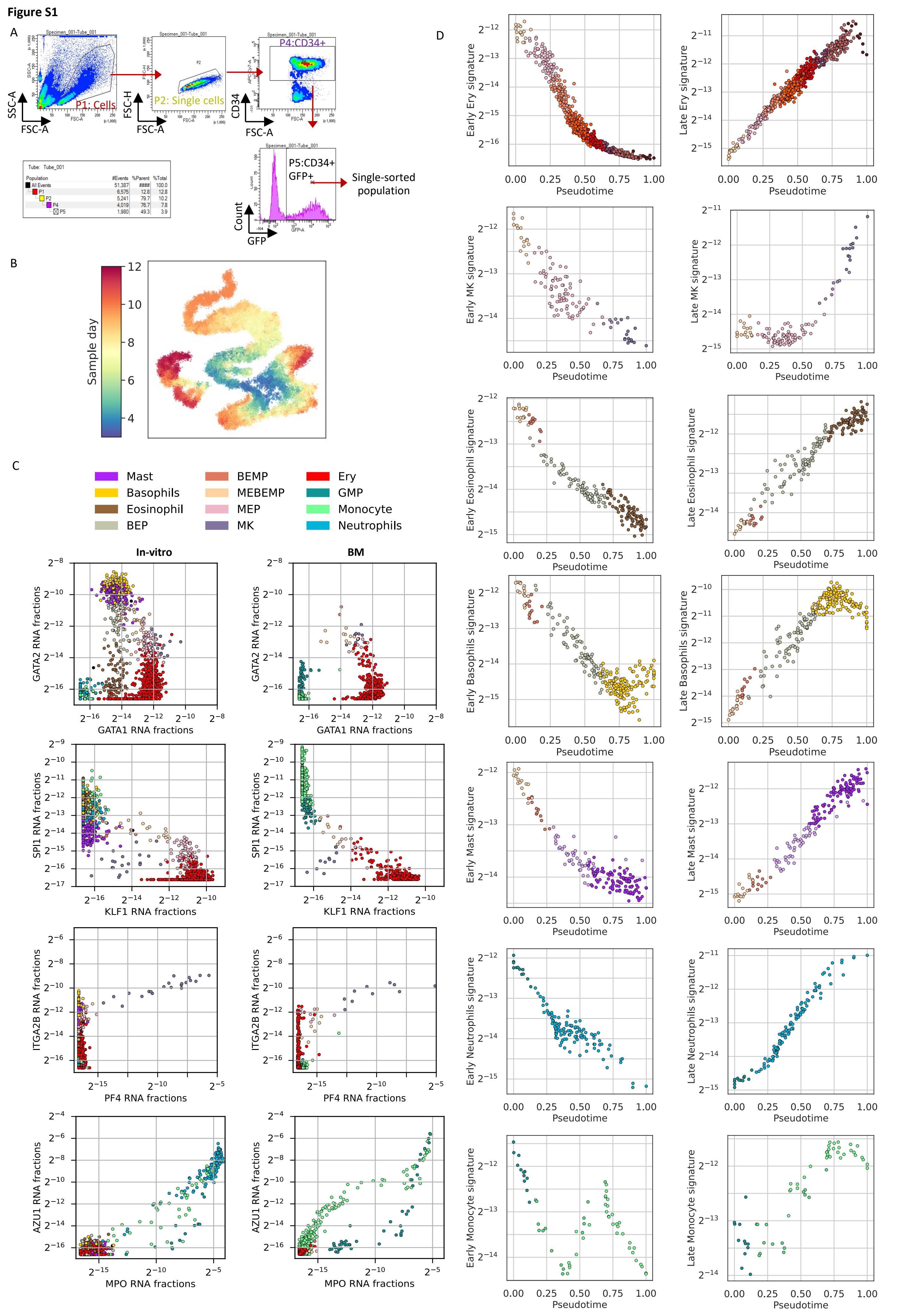

Figure S1
A
P1: Cells
P2: Single cells
P4:CD34+
P5:CD34+ GFP+
Single-sorted population
SSC-A
FSC-A
FSC-H
CD34
Count
GFP
B
Sample day
C
Mast
Basophils
Eosinophil
BEP
BEMP
MEBEMP
MEP
MK
Ery
GMP
Monocyte
Neutrophils
In-vitro
BM
GATA2 RNA fractions
GATA1 RNA fractions
SPI1 RNA fractions
KLF1 RNA fractions
ITGA2B RNA fractions
PF4 RNA fractions
AZU1 RNA fractions
MPO RNA fractions
D
Early Ery signature
Late Ery signature
Early MK signature
Late MK signature
Early Eosinophil signature
Late Eosinophil signature
Early Basophils signature
Late Basophils signature
Early Mast signature
Late Mast signature
Early Neutrophils signature
Late Neutrophils signature
Early Monocyte signature
Late Monocyte signature
Pseudotime

**Fig. S1: Validation of cHSPCTrack states against in vivo hematopoiesis**

A. Gating strategy for the single cell sorting of transduced CD34+ HSPCs
B. Cells sampled day projected over the manifold UMAP.
C. Gene-gene expression plots comparing cHSPCTrack-derived metacells with their BM counterparts from our study and from the HCA. States unmatched to in-vitro states (HSC, MPP, lymphoid) were filtered out. Panels (top to bottom) represent MEP differentiation, MPP differentiation, MK differentiation, and GMP differentiation. Metacells are colored by cHSPCTrack annotations.
D. Early (left) and late (right) modules across pseudotime for all trajectories. Y-axis shows $\log_2$(average fractional expression).

**Figure S2**

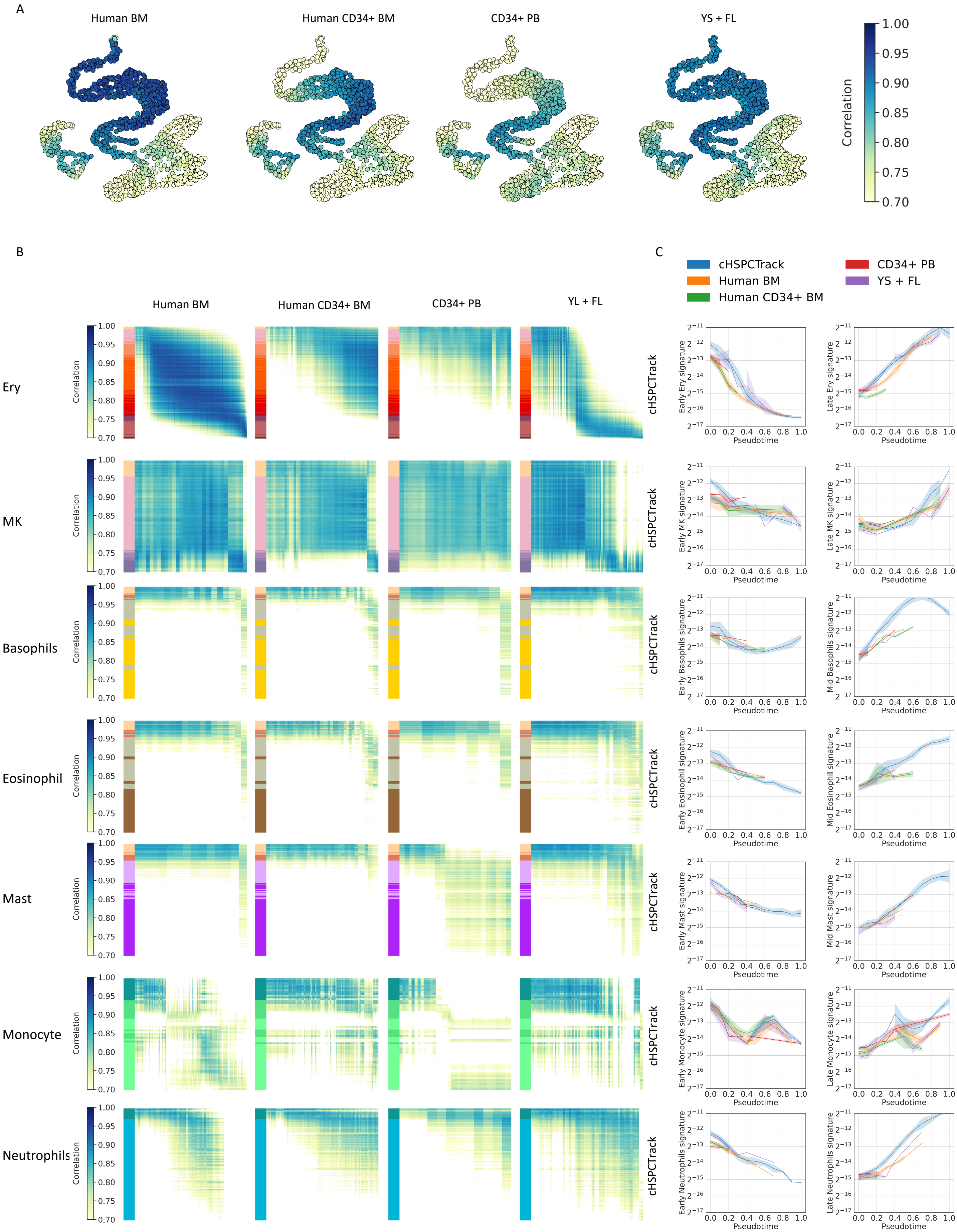
A
Human BM
Human CD34+ BM
CD34+ PB
YS + FL
Correlation
1.00
0.95
0.90
0.85
0.80
0.75
0.70
B
Human BM
Human CD34+ BM
CD34+ PB
YL + FL
Ery
MK
Basophils
Eosinophil
Mast
Monocyte
Neutrophils
C
cHSPCTrack
Human BM
Human CD34+ BM
CD34+ PB
YS + FL
Early Ery signature
Late Ery signature
Early MK signature
Late MK signature
Early Basophils signature
Mid Basophils signature
Early Eosinophil signature
Mid Eosinophil signature
Early Mast signature
Mid Mast signature
Early Monocyte signature
Late Monocyte signature
Early Neutrophils signature
Late Neutrophils signature
Pseudotime

**Fig. S2: Cross-dataset consistency of trajectories and gene programs**

A. UMAP projection of different *in-vivo* datasets metacells over cHSPCTrack manifold showing which cell states appear *in-vivo*. From left to right: Human BM, Human CD34+ BM, CD34+ PB, YL+FB. The ontogeny dataset is included due to its enriched MK representation, improving the resolution of this lineage.

B. Pearson correlation between different trajectories metacells in the *in-vitro* and in-vivo dataset. The color bar shows cell-state identities.

C. Early (left) and mid/late (right) trajectory modules across pseudotime comparing cHSPCTrack dynamics with in-vivo datasets. Y-axis shows $\log_2$(average fractional expression). Trajectories ordered similar to panel B.

**Figure S3**

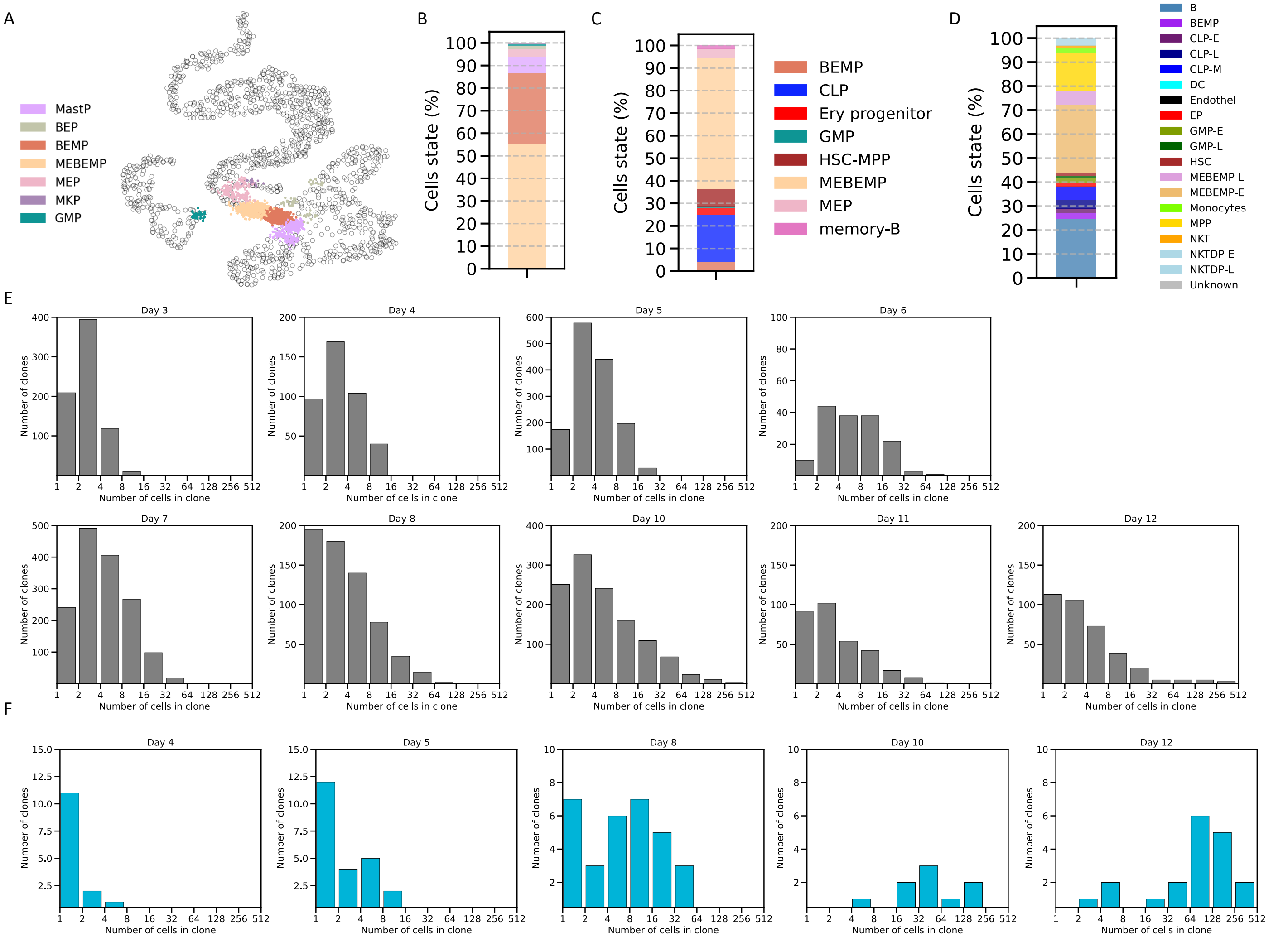


G

| Condition | # Clones | # Cells |
|---|---|---|
| Media I | 878 | 17,058 |
| Media II | 208 | 3,058 |
| Media III | 360 | 9,053 |
| Media IV | 124 | 2,010 |

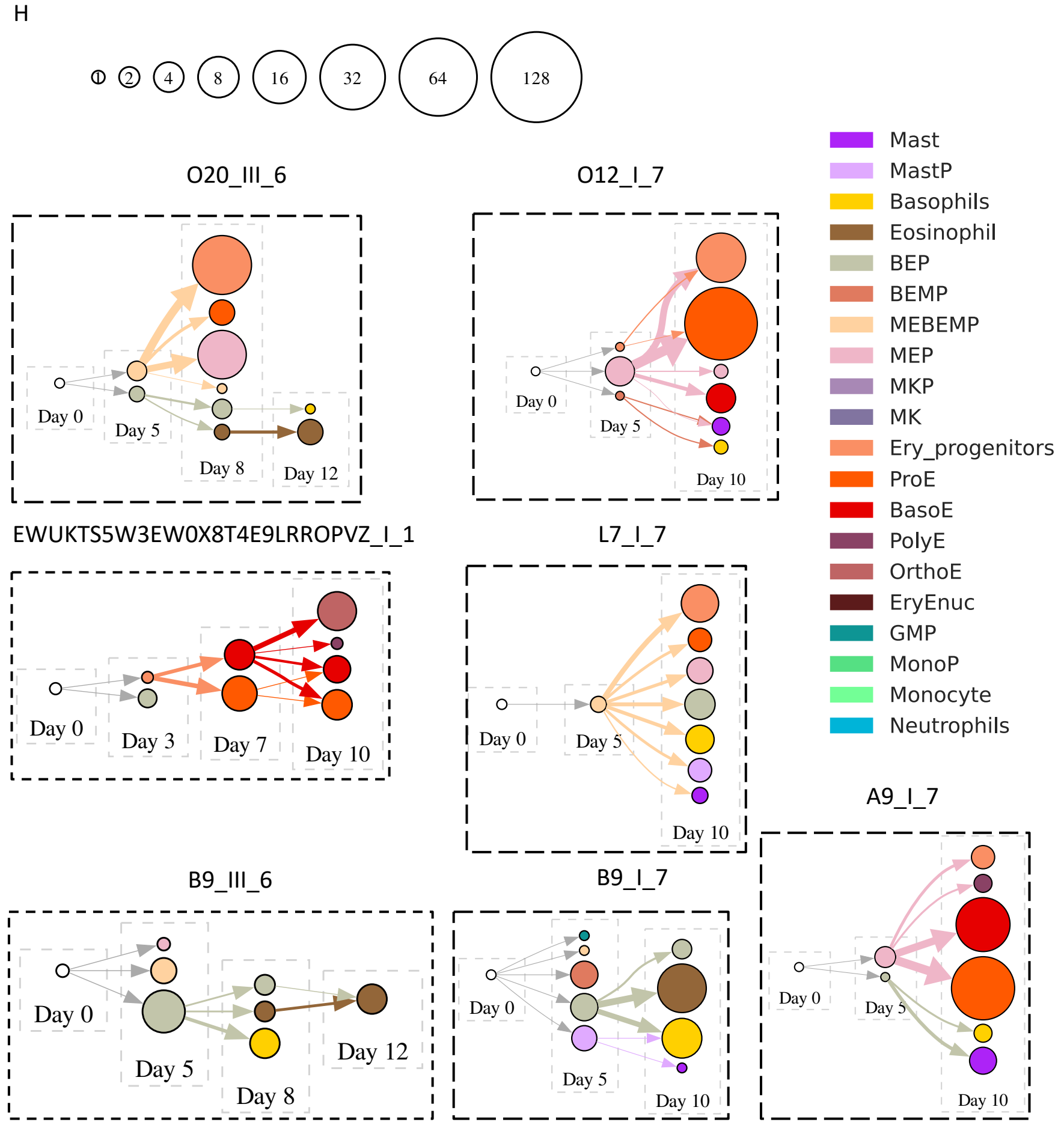


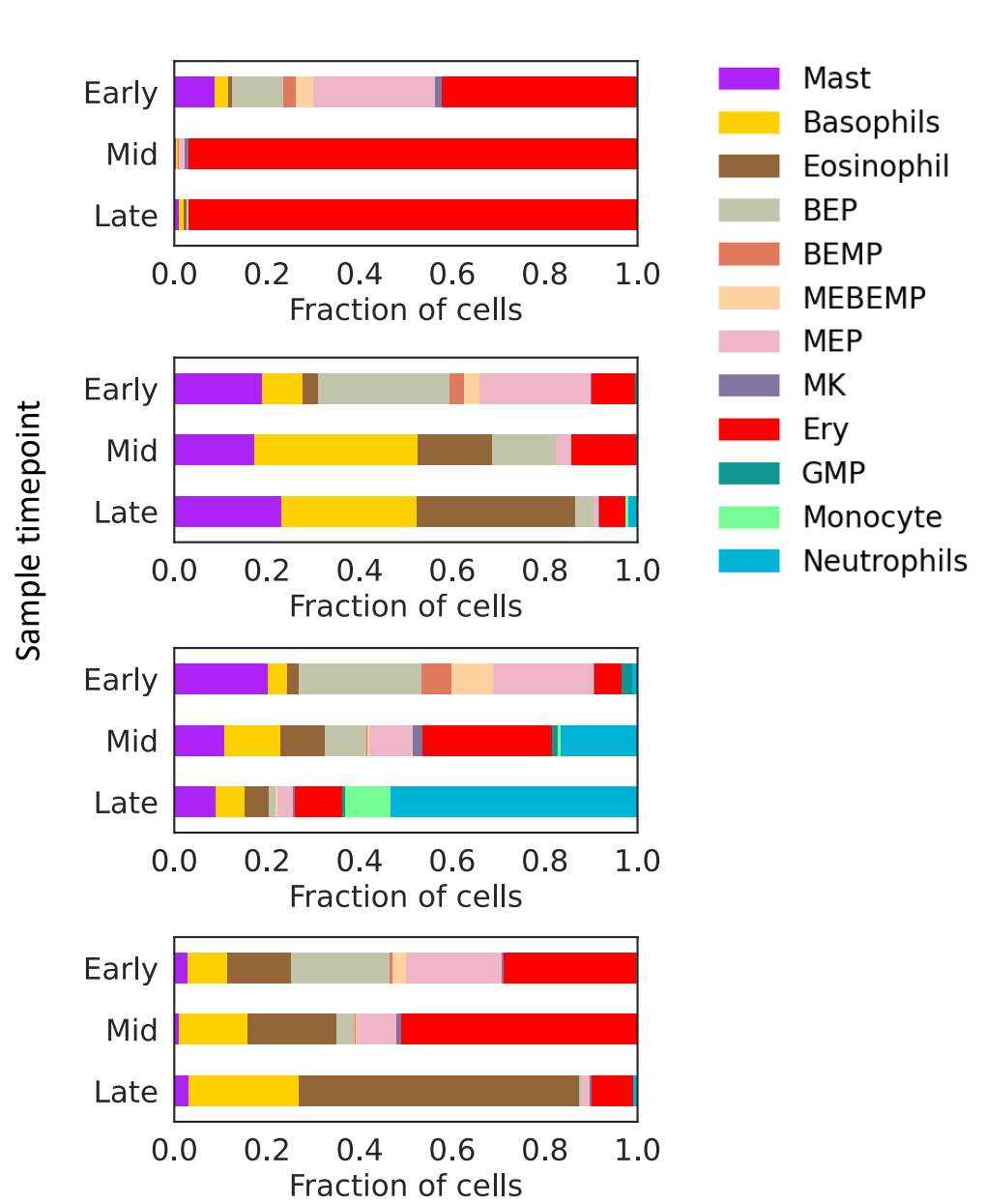

**Fig. S3: Clonal size distribution and dataset composition**

A. UMAP projection of CD34+GFP+ cells over the cHSPCTrack manifold.
B. Percentage of CD34+GFP+ cells state projected over the cHSPCTrack model.
C. Percentage of cultured CD34+ cells state prior to GFP sorting.
D. Percentage of cell states in freshly isolated circulating CD34+ HSPCs. State compositions are shown according to the native annotations and are therefore not directly color-matched to other panels.
E. Distribution of clone sizes across sampling days.
F. Distribution of clone sizes for Neutrophil clones in media III showing rapid proliferation increase at later days.
G. Number of cells and clones in the four main media in which at least 100 clones were sampled in at least 2 timepoints.
H. Clone schematics (circle size = number of cells per state per day; clone names follow *ID_media_replicate* format).
I. Fraction of cells cell state per media, stratified by sampling time bin.

Figure S4

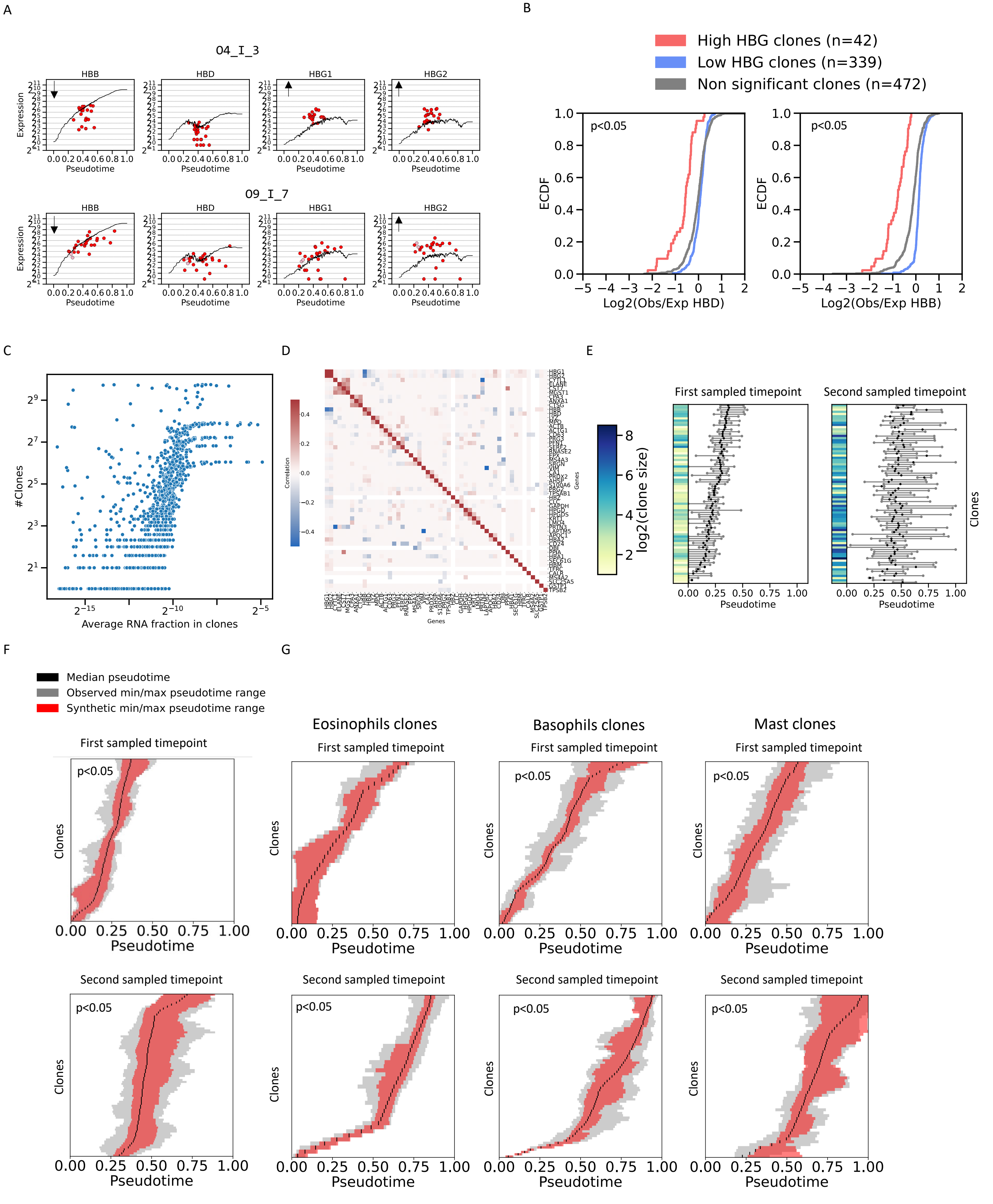

A
04_I_3
09_I_7
HBB
HBD
HBG1
HBG2
Expression
Pseudotime
B
High HBG clones (n=42)
Low HBG clones (n=339)
Non significant clones (n=472)
p<0.05
ECDF
Log2(Obs/Exp HBD)
Log2(Obs/Exp HBB)
C
#Clones
Average RNA fraction in clones
D
Correlation
Genes
E
First sampled timepoint
Second sampled timepoint
log2(clone size)
Clones
Pseudotime
F
Median pseudotime
Observed min/max pseudotime range
Synthetic min/max pseudotime range
First sampled timepoint
Second sampled timepoint
G
Eosinophils clones
Basophils clones
Mast clones

**Fig. S4: Clones exhibit single locus transcriptional memory and stochastic differentiation rates**

A. Examples of clone-specific Beta-locus memory. Gene expression in all downsampled cells of each clone ($log_2$); arrows mark significant over- or under-expression.

B. ECDFs of HBD (left) and HBB (right) $log_2$(observed/expected) expression in clones over/under-expressing HBG, compared with clones without significant HBG deviation.

C. Mean expression of clonal-memory genes vs the number of clones in which they are significant

D. Gene–gene correlation matrix based on clone-level signed co-expression of memory genes.

E. Erythropoiesis pseudotime per clone cells sampled only at two sample days. Each row is a clone, black = median; gray = min/max; left column = cell count. Clones ordered by the first sample day pseudotime median.

F. Observed versus synthetic pseudotime distributions, for erythropoiesis clones sampled at two timepoints.

G. Observed versus synthetic pseudotime distributions, for clones sampled at two timepoints. From left to right: clones containing Eosinophils, Basophils, Mast cells.

**Figure S5**

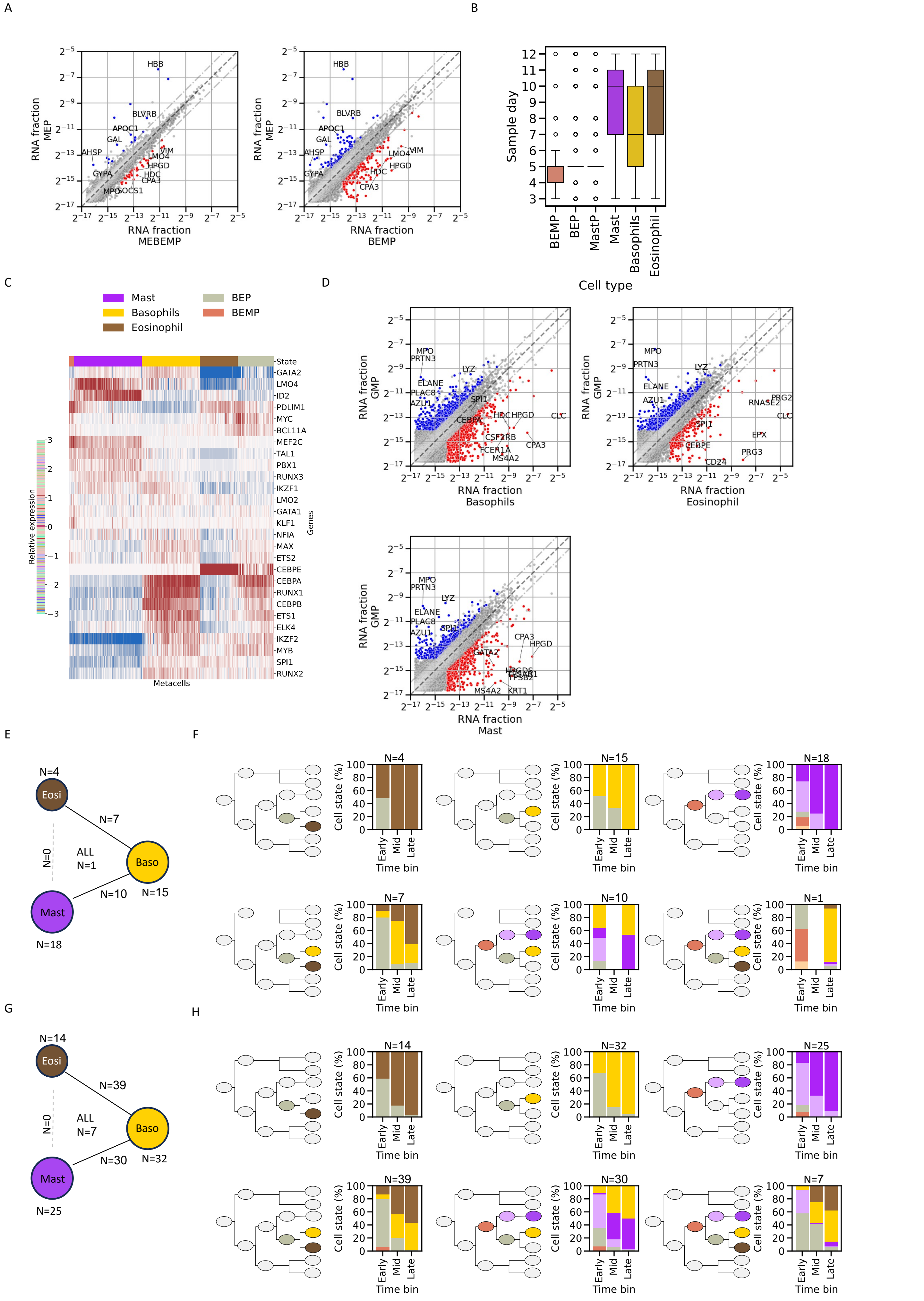
A
RNA fraction MEP
RNA fraction MEBEMP
RNA fraction BEMP
HBB
BLVRB
APOC1
GAL
AHSP
GYPA
MPO
SOCS1
CPA3
HDC
HPGD
LMO4
VIM
B
Sample day
Cell type
BEMP
BEP
MastP
Mast
Basophils
Eosinophil
C
Mast
Basophils
Eosinophil
BEP
BEMP
State
GATA2
LMO4
ID2
PDLIM1
MYC
BCL11A
MEF2C
TAL1
PBX1
RUNX3
IKZF1
LMO2
GATA1
KLF1
NFIA
MAX
ETS2
CEBPE
CEBPA
RUNX1
CEBPB
ETS1
ELK4
IKZF2
MYB
SPI1
RUNX2
Genes
Metacells
Relative expression
D
RNA fraction GMP
RNA fraction Basophils
RNA fraction Eosinophil
RNA fraction Mast
MPO
PRTN3
LYZ
ELANE
PLAC8
AZU1
SPI1
CEBPA
HDC
HPGD
CLC
CSF2RB
CPA3
FCER1A
MS4A2
RNASE2
PRG2
EPX
PRG3
CEBPE
CD24
GATA2
KRT1
E
Eosi
Baso
Mast
N=4
N=7
N=0
ALL
N=1
N=10
N=15
N=18
F
Cell state (%)
Time bin
Early
Mid
Late
N=4
N=15
N=18
N=7
N=10
N=1
G
Eosi
Baso
Mast
N=14
N=39
N=0
ALL
N=7
N=30
N=32
N=25
H
Cell state (%)
Time bin
Early
Mid
Late
N=14
N=32
N=25
N=39
N=30
N=7

**Fig. S5: Transcriptional dynamics and clonal structure of BEMP differentiation**

A. Gene expression comparison between MEBEMP and MEP (left) and BEMP and MEP (right). Each point shows the absolute expression of a gene ($\log_2$ UMI fraction). Diagonal dashed lines mark 2-fold differences. Blue/red denote significant y/x-axis overexpression.

B. Boxplot showing sample day distribution of BEMP lineage cells.

C. Heatmap of TF (rows) per BEMP trajectory cell-state metacells (columns).

D. Gene expression comparison between (clockwise): Basophils, Eosinophils and Mast vs GMP, displayed as in panel A.

E. Schematic of BEMP-only clones in media I. N indicates clone counts adjacent to nodes (single-fate), along edges (two-fate), and at the center (all fates).

F. BEMP-only clone (media I) differentiation paths (left) with corresponding transcriptional-state distributions over time bins (right) for each terminal state combination. Top: single-fate clones; bottom: multi-fate clones. *N*= matches panel E.

G. Schematic of BEMP-only clones in media II displayed as in panel E.

H. BEMP-only clone (media II) differentiation paths (left) with corresponding transcriptional-state distributions over time bins (right) for each terminal state combination. Top: single-fate clones; bottom: multi-fate clones. *N*= matches panel G.

**Figure S6**

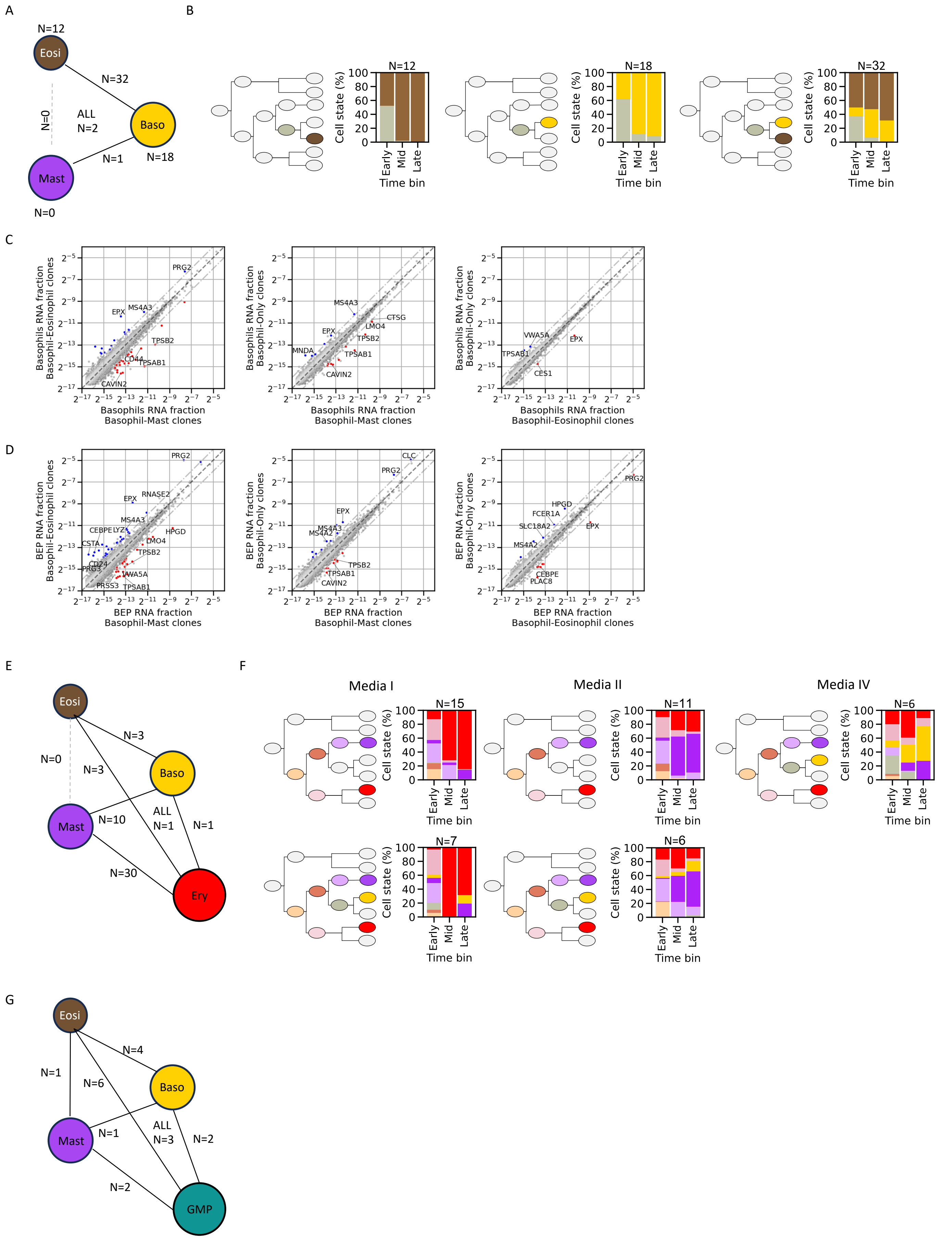
A
B
N=12
Eosi
N=32
N=0
ALL
N=2
Baso
N=1
N=18
Mast
N=0
Cell state (%)
Early
Mid
Late
Time bin
C
Basophils RNA fraction
Basophil-Eosinophil clones
Basophil-Only clones
Basophil-Mast clones
PRG2
MS4A3
EPX
TPSB2
CD44
TPSAB1
CAVIN2
CTSG
LMO4
MNDA
VWA5A
CES1
D
BEP RNA fraction
RNASE2
CEBPE
LYZ
HPGD
CSTA
CD24
PRG3
PRSS3
CLC
MS4A2
FCER1A
SLC18A2
PLAC8
E
F
Media I
Media II
Media IV
N=15
N=11
N=6
N=7
N=3
N=10
N=30
Ery
G
N=4
N=6
N=3
GMP

**Fig. S6: Modulation of BEMP lineage outputs by paired clonal states**

A. Schematic of BEMP-only clones in media IV. N indicates clone counts adjacent to nodes (single-fate), along edges (two-fate), and at the center (all fates).

B. BEMP-only clone (media IV) differentiation paths (left) with corresponding transcriptional-state distributions over time bins (right) for each terminal state combination. Top: single-fate clones; bottom: multi-fate clones. *N*= matches panel A.

C. Gene expression comparison between Basophil cells depended on clone output. Each point shows the absolute expression of a gene ($\log_2$ UMI fraction). Diagonal dashed lines mark 2-fold differences. Blue/red denotes significant y/x-axis overexpression.

D. Gene expression comparison between BEP cells dependent on clone output. Each point shows the absolute expression of a gene ($\log_2$ UMI fraction). Diagonal dashed lines mark 2-fold differences. Blue/red denotes significant y/x-axis overexpression.

E. Schematic of BEMP-Ery clones in media III displayed as in panel A.

F. Media I, II, IV BEMP-Ery clone differentiation path (left) with corresponding transcriptional-state distributions over time bins (right) for each terminal state combination. Clones combinations with enough clones (>2) are displayed.

G. Schematic of BEMP-GMP clones in media III displayed as in panel A.
